# Discovery of mCMV280: An Oral Ectoparasiticide in the Isoxazoline Class with Reduced Mammalian Brain Exposure

**DOI:** 10.1021/acs.jmedchem.5c03776

**Published:** 2026-03-03

**Authors:** Sarah E. McComic, Katy B. Wilson, Zhilin Li, Jeffrey Chen, Frank Weiss, Lirui Song, Wrickban Mazumdar, Curt A. Dvorak, Jason Brittain, Kyoung-Jin Lee, Shuangwei Li, Sean B. Joseph, Case W. McNamara, Martijn W. Vos, Avinash Sheshachalam, Alex Inácio, Koen J. Dechering, Arnab K. Chatterjee, Daniel R. Swale

**Affiliations:** † Department of Entomology and Nematology, Emerging Pathogens Institute, 3463 University of Florida, Gainesville, Florida 32610, United States; ‡ Calibr-Skaggs Institute for Innovative Medicines at Scripps Research, La Jolla, California 92037, United States; § 561119 TropIQ Health Sciences, Transistorweg 5, 6534 AT Nijmegen, The Netherlands; ∥ QM Diagnostics, Transistorweg 5, 6534 AT Nijmegen, The Netherlands

## Abstract

Vector-borne diseases represent a significant global health concern, and effective vector control in animals often involves using orally administered drugs that kill arthropod vectors of human pathogens. Isoxazoline ectoparasiticides may have promise in humans if they can be optimized for safe use due to their selectivity for invertebrate over mammalian ion channels. Yet, isoxazolines can cause neurological side effects due to their ability to cross the blood brain barrier, and thus, we synthesized novel isoxazolines with improved physiochemical properties to reduce brain exposure without reducing toxicity to arthropod pests. Our medicinal chemistry campaigns led to the discovery of lead compound mCMV280 that is 3× more toxic to ticks and equitoxic to mosquitoes, with an ∼5× reduction in mammalian brain exposure and an ∼8× lower brain-to-plasma ratio compared to fluralaner. These findings highlight the promise of new isoxazoline scaffolds for safer and more effective drug-based vector control strategies in humans.

## Graphical abstract

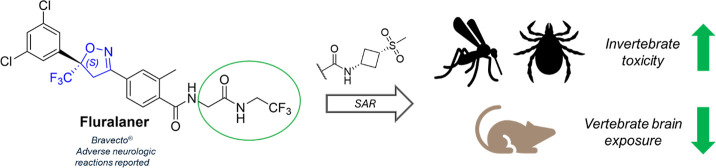

## Introduction

Mosquitoes are vectors for numerous pathogens that cause devastating diseases of relevance to global health, such as malaria, dengue fever, and lymphatic filariasis that infect hundreds of millions of people each year causing significant morbidity and mortality globally.^1,2^ In addition to mosquito-borne diseases, tick-borne diseases (TBDs) in humans have steadily increased over the past 10 years across the America and Europe, accounting for >75% of the nearly 650,000 cases of vector-borne diseases reported in the United States during 2004–2016.^3^ If accurately diagnosed early, the major TBDs such as Lyme disease and tick-borne rickettsial diseases (TBRD) can be effectively treated with antibiotics, but feeding ticks often go unnoticed and the nondescript flu-like symptoms often go undiagnosed.^4^ This results in significant disease progression and increased potential for long-term post-treatment complications of the infection.^5–7^ To address this health burden, we aimed to identify novel chemical scaffolds that can reduce transmission of tick-vectored pathogens by inducing toxicity in less than 12 h, which is the minimum feeding time required for transmission of some bacterial pathogens.^6^

Chemical pesticides continue to be the mainstay for the control of ectoparasites and reduction of vector-borne diseases. Yet, overuse of insecticides/acaricides has driven the evolution of widespread resistance that has reduced their efficacy,^8^ which highlights the need to develop or optimize chemical classes that are not sensitive to established mechanisms of resistance and can be delivered as an ectoparasiticide. Efforts have been made to establish platforms for the discovery of novel insecticides,^9–12^ but the developmental pipeline from discovery to commercialization is long and expensive. Therefore, modification of existing pesticidal classes has become a viable approach for novel insecticide development.^13–16^ The isoxazoline class of compounds are a group of selective and broadly potent invertebrate GABA and l-glutamate-gated chloride channel inhibitors currently approved for flea and tick control in companion animals (Figure 1).^17–19^ Isoxazolines are highly bound by plasma proteins and are very slowly cleared, which results in a long half-life and reduces the number of doses required for effective arthropod control. Given these characteristics, we, and others,^20,21^ envisioned the isoxazolines as attractive candidates for vector control in countries with endemic mosquito-vector disease like malaria.^22^ Oral dosage of a long-acting ectoparasiticide would provide an additional tool to reduce vector populations that would act on both indoor and outdoor mosquitoes and complement the currently available tools.^23^ Further, the prophylactic action of a rapid kill oral ectoparasiticide could provide long-term protection of humans from TBDs, such as Lyme disease and TBRD. However, in 2018, the FDA released a statement^24^ warning pet owners of the potential adverse neurological events that had been reported in dogs and cats, including ataxia, muscle tremors, and seizures, which raised concern for the potential of isoxazolines to be used in humans for control of arthropod-vectored pathogens.

**Figure 1:**
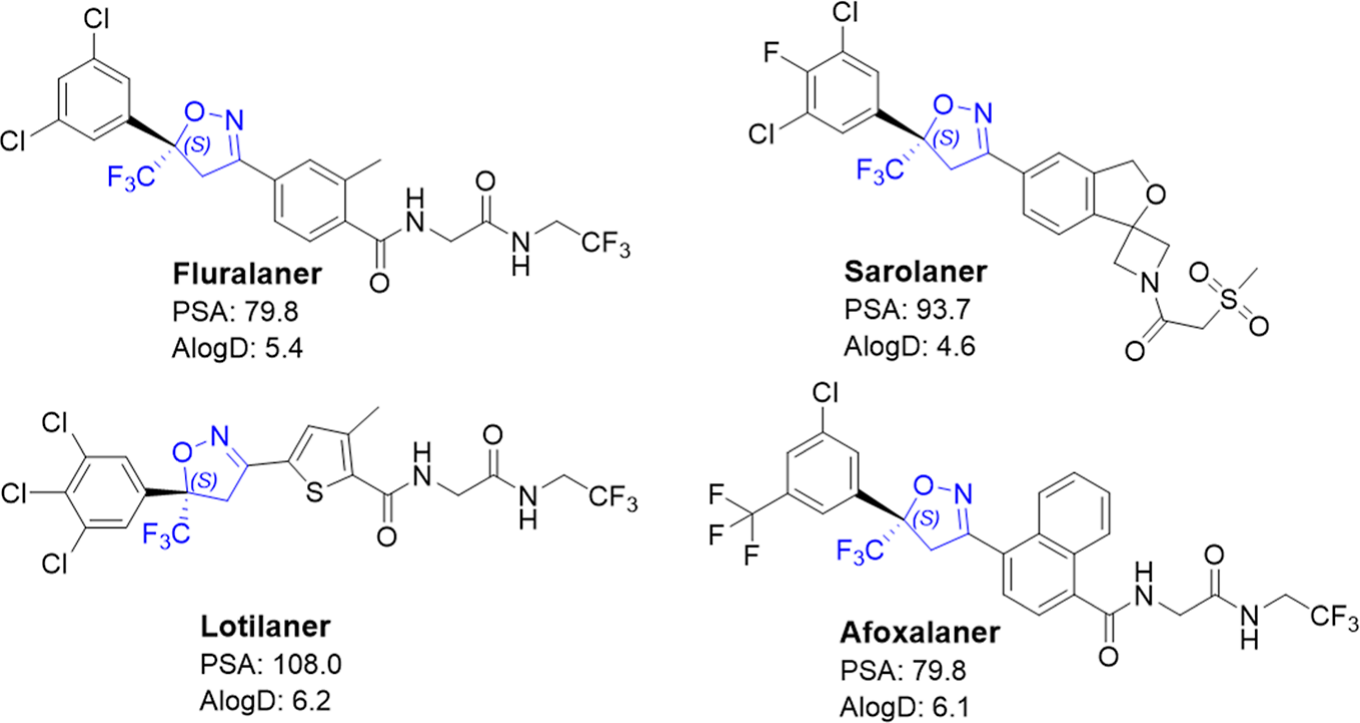
The marketed isoxazolines currently approved for use in companion animals for pest control (ticks, flees, and mites). All compounds share the common isoxazoline core with a quaternary carbon bearing a CF_3_ group. Although some of these treatments are dosed as a racemic mixture, the enantiomer with (*S*) configuration in the isoxazoline ring is generally understood to be the active ingredient. These compounds typically have high AlogD and are highly bound by plasma proteins. PSA = polar surface area.

Therefore, significant chemical optimization is needed to overcome potential neurotoxicity of commercialized isoxazolines. Herein, we describe a structure–activity relationship (SAR) campaign to develop a novel endectocide with reduced brain exposure in mammalian model systems to mitigate potential mammalian neurotoxicity of isoxazolines while maintaining a high toxicity to mosquitoes and ticks.

## Results and Discussion

### Mosquitocidal Effects

To assess the suitability of commercial isoxazoline compounds for mosquito vector control, we first compared the toxicity profiles against key insect species using a standardized assay. We profiled commercialized isoxazolines in the standard membrane feeding assay (SMFA) against two different mosquito species: *Anopheles stephensi* and *Aedes aegypti*. The rank order of potencies against *An. stephensi* was fluralaner = afoxolaner > sarolaner > lotilaner. Fluralaner and afoxolaner were the most potent compounds to *An. stephensi* with LC_50_ values of 39 nM (95% CI: 28–50; Hillslope: 3.5; *r* ^2^: 0.98) and 51 nM (95% CI: 38–77; Hillslope: 3.2; *r* ^2^: 0.97) at 24 h, which were not significantly different from each other (Table 1). Lotilaner was the least potent, with an LC_50_ of 198 nM (95% CI: 165–236; Hillslope: 1.8; *r* ^2^: 0.98) (Table 1). The rank order of toxicity against *Ae. aegypti* was slightly different compared to the rank order of *An. stephensi* with fluralaner = sarolaner > afoxolaner > lotilaner. The fluralaner LC_50_ was found to be 29 nM (95% CI: 22–36; Hillslope: 4.7; *r* ^2^: 0.96), which was not significantly different when compared to the toxicity against *An. stephensi*. The sarolaner LC_50_ (38 nM, 95% CI: 25–52, Hillslope: 1.5, *r* ^2^: 0.96) against *Ae. aegypti* was not significantly different when compared to the potency of fluralaner against *Ae. aegypti* but was significantly different from sarolaner toxicity to *An. stephensi* (Table 1). Afoxolaner and lotilaner LC_50_ values against *Ae. aegypti* were not significantly different from the LC_50_ values against *An. stephensi*. Due to the relative lack of statistical differences between the LC_50_ values of *An. stephensi* and *Ae. aegypti*, these two species were treated interchangeably for downstream SAR analyses.

**Table 1:** Profiling Data for the *S* Enantiomer of Marketed Isoxazolines against Ticks and Mosquitoes^a^,^b^

treatment group	lotilaner	afoxolaner	fluralaner	sarolaner
*An. stephensi* 24 h LC_50_ (nM	198 (A, a)	51 (B, a)	39 (B, a)	90 (C, a)
*Ae. aegypti* 24 h LC_50_ (nM)	246 (A, a)	102 (B, b)	29 (C, a)	38 (C, b)
*Am. americanum*	408 (A, a)	55 (B, a)	170 (C, a)	100% at 200 nM
12 h; 24 h LC_50_ (nM)	139 (A, b)	25 (B, a)	48 (B, b)	
MDCK MDR1 P_app_ A-B/B-A (10^–6^ cm/s)	0.65/1.55	1.2/<1	6.12/3.95	<1/<1
mouse brain *K*_p,uu_ (@ *C*_max,brain_ and AUC_all_)	0.09/0.06	0.025/0.004	0.017/0.016	1.19/1.13
mouse brain:plasma ratio (@ *C*_max,brain_ and AUC_all_)	0.49/0.30	0.025/0.004	0.95/0.90 (ms)	1.19/1.13
			0.95 (terminal, dog)	
			1.16 (terminal, cyno)	

a LC_50_ values were determined using a nonlinear regression (variable-slope) analysis on GraphPad Prism 10 (San Diego, CA, USA). Each CRC consisted of 6–8 concentrations with 10 individuals per concentration, and each concentration was replicated at least 5 times using different batches of arthropods, which results in 50 individual mosquitoes per concentration. Letters designate statistical significance where chemicals not labeled by the same capital letter represent significance at *P* < 0.05 for different chemical classes for the same species, as determined by one-way ANOVA with Tukey’s post-test. Lowercase letters compare the same chemical across mosquito species or compares 12 to 24 h toxicity for ticks, and those not labeled by the same lowercase letter represent statistical significance (*P* < 0.05) as determined by an unpaired *t*-test

b LC_50_ = 50% lethal concentration, MDCK MDR1 = Madin–Darby canine kidney Multidrug Resistance 1 permeability assay, P_app_ = apparent permeability, A-B = apical-to-basolateral direction, B-A = basolateral-to-apical direction, *K*_p,uu_ = unbound brain-to-blood concentration ratio, *C*_max,brain_ = maximum concentration in the brain, and AUC_all_ = area under the curve from time zero to the last time point.

The correlation between in vitro assay outcomes and in vivo efficacy was then assessed by feeding mosquitoes on mice orally dosed with the ectoparasiticides. Previously, in vivo studies with ivermectin have demonstrated prolonged killing effects despite low plasma concentrations, presumably due to distribution of the compound outside the blood compartment.^25^ To study the in vitro–in vivo correlation for the class of isoxazolines, an efficacy study was conducted in mice with *S*-fluralaner and *S*-afoxolaner. Mice were dosed by oral gavage with 1, 4, 16, or 64 mg/kg of the compound, and blood concentrations were monitored over time. *An. stephensi* mosquitoes were fed on the mice at regular time intervals, and mosquito mortality was assessed 48 h post feeding. As expected, increased concentrations of isoxazolines in the blood led to a decreased survival of mosquitoes (Figure 2A,B) with LC_50_ values of 37 and 15 nM for afoxolaner and fluralaner, respectively. Importantly, this correlates very well with the IC_50_ values of 25 and 29 nM at 48 h after feeding established in SMFAs (Figure 2C,D). These data support the validity of the SMFA for the initial assessment of pharmacodynamics in relation to blood concentration.

**Figure 2:**
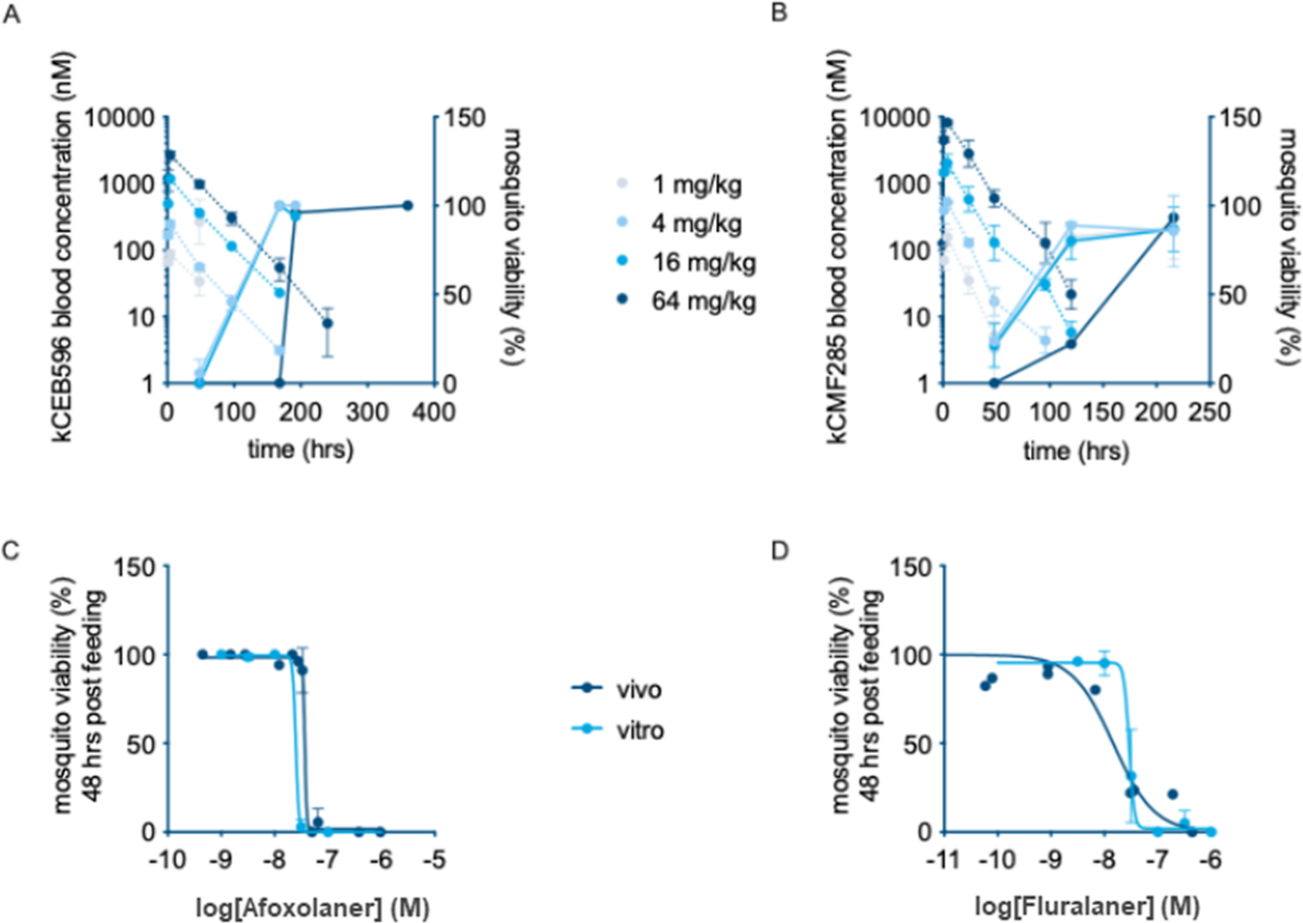
In vitro–in vivo correlation of isoxazoline toxicity to mosquitoes after oral treatment of mice. Mice were dosed with 1–64 mg/kg afoxolaner or fluralaner orally, and circulating compound levels and mosquitocidal activity were monitored in time. (A, B): Blood concentrations (dashed lines, left *y*-axis) and mosquito viability data (solid lines, right *y*-axis) for afoxolaner (A) and fluralaner (B). The figures show compound levels in whole blood and mosquitocidal activity as monitored by mosquito direct skin feeding. Mosquito mortality was evaluated 48 h post feeding and plotted at the time point of feeding. Each treatment group had two mice, and error bars indicate standard deviations. (C, D): comparison of in vitro and in vivo dose–response relationships. The figure shows in vitro and in vivo blood concentration versus mosquito viability determined 48 h post feeding for afoxolaner (C) and fluralaner (D), with each data point representing mean (*n* = 5) and error bars representing SD.

### Systemic Acaricidal Effects

The acaricidal effects against lone star ticks (*Am. americanum*) was determined for the commercialized isoxazolines at 12 and 24 h because more rapid toxicity (within the first 16 h) is more likely to prevent the transmission of tick-borne pathogens.^6^ The rank order of toxicity was found to be afoxolaner = sarolaner > fluralaner > lotilaner at 12 h and afoxolaner = sarolaner = fluralaner > lotilaner at 24 h (Table 1). Afoxolaner and sarolaner were equitoxic and were the most toxic compounds tested at 12 h with LC_50_ values of 55 nM (95% CI: 22–111 nM, Hillslope: 1.1, *R* ^2^: 0.94) and 55 nM (95% CI: 36–75 nM, Hillslope: 2.2, *R* ^2^: 0.99), respectively. Afoxolaner toxicity was significantly (*P* < 0.05) better than fluralaner LC_50_ at 12 h but was not significantly different at 24 h, where no significant difference in LC_50_ values was observed for afoxolaner, sarolaner, and fluralaner with LC_50_ values ranging from 17 to 48 nM. Lotilaner was the least toxic with an LC_50_ of 408 nM (95% CI: 356–473, Hillslope: 2.4, *R* ^2^: 0.99) at 12 h but toxicity increased by approximately 3-fold at 24 h when compared to 12 h, which was a statistically significant (*P* < 0.05) change from 12 to 24 h post ingestion (Table 1).

### Pharmacokinetic Studies of Isoxazolines in Mammals

Because of the noted adverse neurological effects of this class of compounds, oral PK studies on commercialized isoxazolines were conducted in mice, and the plasma and brain concentrations were measured at select time points over a 24 h period. Fluralaner and sarolaner had an average blood–plasma ratio (B/P) of ∼1. The B/P of lotilaner was 0.5 and afoxolaner had the lowest B/P of 0.025 when B/P was calculated at *C*_max_ in the brain (Table 1). Plasma protein binding (PPB) and brain homogenate binding (BHB) data were also collected to calculate the *K*_p,uu_ values (Table 1). Both *K*_p,uu_ and B/P were used to evaluate CNS exposure because the high level of binding in the plasma and brain led to lower confidence in the *K*_p,uu_ calculations, with binding values often greater than 99.9%. The difference in B/P ratios between species was also evaluated with fluralaner when it was dosed orally during beagle dog (*Canis familiariz*) and cynomolgus monkey (*Macaca fascicularis*) PK studies, and terminal brain concentrations were then measured (Table 1). These results show consistent B/P ratios across species, with 0.95 for mice, 0.95 for beagle dogs, and 1.16 for cyno monkeys.

### Reducing Brain Exposure to Mammals

Continuing with the SAR campaign, analogs of fluralaner were designed with the goal of reducing brain exposure while maintaining arthropod toxicity and also introducing structural uniqueness from existing isoxazolines. New analogs were tested in the SMFA with *An. stephensi* and *Ae. aegypti* mosquitoes, and compounds eliciting 80% or higher mortality at a discriminate dose (200 nM) were dosed orally in mouse brain PK studies. The MDCK MDR1 permeability assay was also used to help predict the efflux properties of new analogs and identify compounds that might have reduced brain exposure due to active efflux. The SAR strategy to reduce CNS penetration was to increase polar surface area (PSA), the number of heavy items, the molecular weight (MW), and the number of hydrogen bond donors (HBD) and reduce logD.

### Terminal Phenyl Ring Modifications and Activity

There are four distinct regions on the isoxazoline structure where chemical modifications can be made: the two aryl rings, the isooxazoline ring, and the amide group (Figure 3). The general synthesis scheme to synthesize these fluorinaner analogs is outlined in Scheme 1, with the key step being step 2 where the 3 + 2 cyclization forms the isooxazoline ring as a racemic mixture. Each enantiomer of the final compounds was tested in the SMFA, and the active compound was confirmed to be the (*S*) enantiomer. Synthesis of numerous analogs (only key examples shown) showed that SAR on the terminal aryl ring is typically tight with meta- and para-halogens and some other electron withdrawing groups (CF_3_) being tolerated. Substitution of one of the Cl groups in fluralaner to a CN group gives a decrease in potency but a reduction in brain exposure in an oral mouse PK study (Table 2, mCMN879). Although some more drastic modifications on the phenyl ring helped reduce brain exposure, they also caused loss of ectoparasiticidal activity so the phenyl ring was disregarded for further modifications.

**Figure 3:**
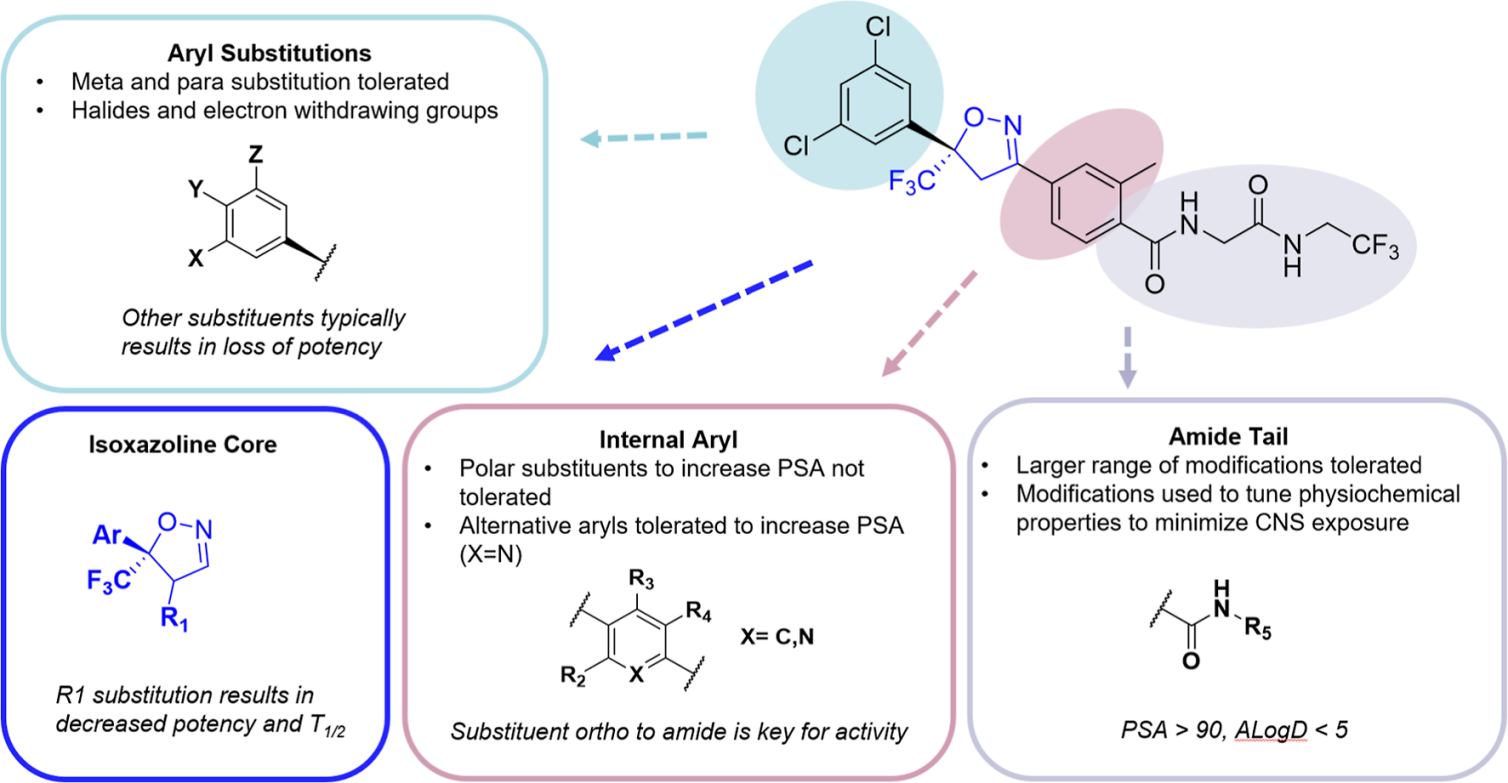
SAR strategy leading to mCMV280 as late lead with balanced potency and desired physicochemical properties. Reduce brain exposure by increasing PSA, MW, and number of heavy atoms while maintaining potency in the SMFA assay and reducing off-target liabilities. Prioritize novel compounds with optimal properties (PSA > 90, ALogD< 5). SAR = structure activity relationship, MW = molecular weight, PSA = polar surface area, and SMFA = standard membrane feeding assay.

**Scheme 1:**
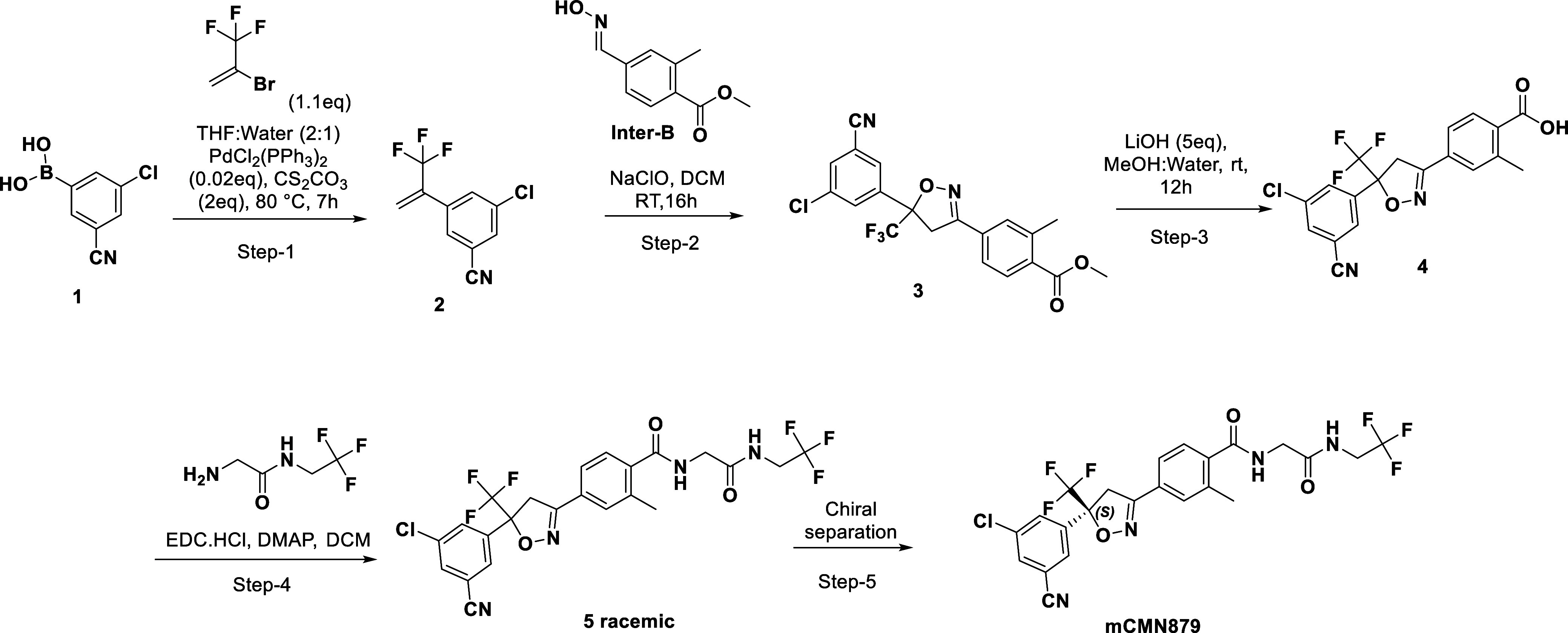
General Synthetic Scheme for Synthesis of Isoxazoline Compounds^a^

a Synthetic route to make racemic isooxazoline compounds. Synthesis of key intermediate **B** is outlined in the Supporting Information.

**Table 2:** SAR Profile of Fluralaner Analogs^a^

analog	*A* log *D*	PSA	*An. stephensi* LC_50_ at 24, 48 h (nM)	mPPB (%)	mBHB (%)	MDCK MDR1 *P*_app_ A-B/B-A (10^–6^cm/s)	Ms B/P (AUC)	Ms B/P (*C*_max,brain_)	Ms *K*_p,uu_
mCMN879	4.8	104	274, 173	99.99	99.8	10.3/28.3	0.045	0.04	0.8
mCMN882	5.7	79.8	44, 26	>99.99	>99.99	5.0/18.6	0.4	0.6	0.6
mCMF883	6.4	79.8	110, 66	97.34	>99.99	2.4/6.2	0.76	0.75	0.0025
mCMO213	4.7	92.7	100% at 200 nM	98.73	>99.99	5.5/9.8	2.75	3.33	0.026
mCMO036	5.9	79.8	91, 51	99.8	99.99	2.3/4.5	0.16	0.16	0.0074
mCMN570	5.1	96.5	134, 125	96.5	>99.99	0.1/0.9	0.27	0.29	0.0006

a PSA = polar surface area, LC_50_ = 50% lethal concentration, mPPB = mouse PPB, mBHB = mouse BHB, MDCK MDR1 = Madin–Darby canine kidney Multidrug Resistance 1 permeability assay, *P*_app_ = apparent permeability, A-B = apical-to-basolateral direction, B-A = basolateral-to-apical direction, Ms = mouse, B/*P* = brain-to-plasma ratio, AUC = area under the curve, *C*_max,brain_ = maximum concentration in the brain, and *K*_p,uu_ = unbound brain-to-blood concentration ratio.

### Isoxazoline Modifications and Corresponding Activity

Modifications on the isoxazoline moiety were explored to introduce novelty to the chemical series, with a surprising SAR uncovered at R1 (Figure 3). Substitution at R1 with halogens (F, Cl, and Br) and alkyls (methyl, dimethyl, and ethyl) showed poor activity relative to fluralaner; however, the allyl compound mCMF883 (Figure 4) resulted in reasonable toxicity to mosquitoes (LC_50_ = 110 nM) (Table 2). Although the SAR here was intriguing, this direction was put on hold due to the increased clearance, lower exposure, and a shorter half-life observed in mouse PK studies.

**Figure 4:**
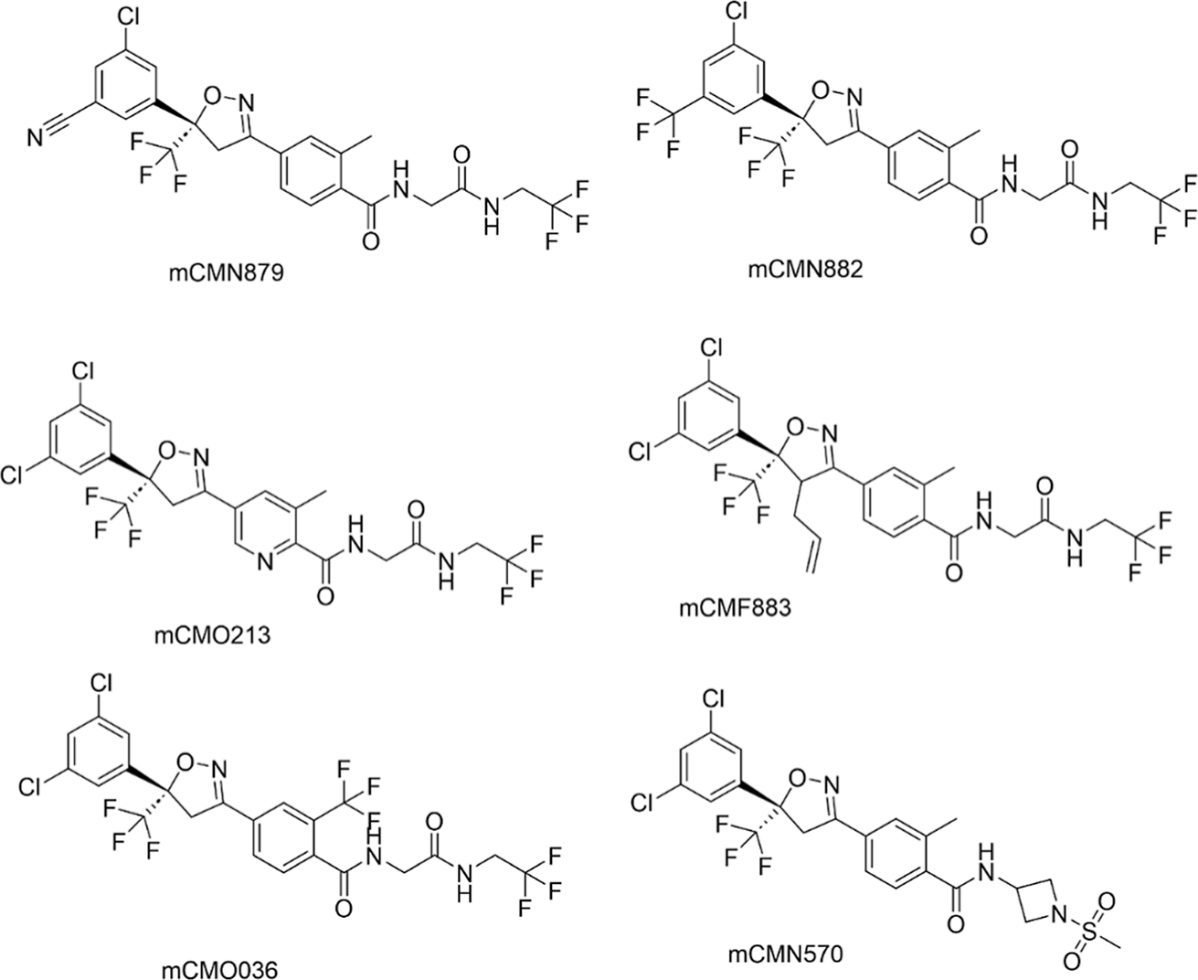
Structures of initial SAR campaign to reduce mammalian CNS penetration.

### Internal Aryl Ring Substitutions Necessary for Activity

During SAR exploration of the internal aryl ring, it was found that ortho substitution adjacent to the amide is necessary for the activity. Substitution of the ortho methyl with alternatives like CF_3_ and SF_5_ was tolerated but did not provide substantial improvement in CNS restriction based on mouse PK (Table 2). Exchanging the phenyl ring for a pyridine ring in mCMO213 (Figure 4) provided good potency in mosquito and tick ingestion assays but gave the highest B/P observed among new analogs with a B/P of 2.75.

### Amide Tail Modifications Provided the Best SAR

The greatest success in maintaining arthropod toxicity with a reduction in mammalian CNS exposure was achieved through modifications of the amide tail. Changes here allowed for increased polarity while maintaining arthropod potency, with the most promising compound in this SAR series, mCMN570 (Figure 4), containing a sulfonamide group that increased the PSA above 90 and reduced AlogD to 5.1 (Table 2). mCMN570 has a B/P of 0.29 and the best *K*_p,uu_ of 0.0006. Further SAR exploration was then performed with the amide tail with the goal to improve potency and novelty, as the sulfonamide group of mCMN570 was previously described in the patent literature.^26^

### Mosquito and Tick Toxicity of Amide Tail-Substituted Isoxazolines

The 24 h mortality was assessed for amide SAR compounds at 200 nM in *Ae. aegypti* mosquitoes. Compounds eliciting 100% mortality at this concentration were further evaluated at 50 nM. Additionally, the same compounds were tested in ticks at 200 nM using an artificial membrane feeding assay, which provided precise control over the timing of feeding after attachment.^12,27^

The optimization of a compound with broad vector potency was desired for applications from malaria to Lyme disease. Considering this, the four-membered azetidine ring of mCMN570 was expanded to five-membered ring structures (mCMV092 and mCMV074) to further improve the potency and stability. However, this modification resulted in reduced toxicity against mosquitoes with 58% and 27% mortality at 200 nM, respectively (Table 3). Alternatively, nitrogen removal from the azetidine ring produced the cyclobutyl analogs of mCMN570, incorporating a sulfonyl group instead of the sulfonamide group. Both the *cis* and *trans* analogs of the mCMN570 modification (mCMV280 and mCMV304) demonstrated 100% mortality at 200 nM in mosquitoes 24 h post feeding. However, the *trans* analog showed only 51% mortality at 50 nM, whereas mCMV280 maintained 100% mortality at 50 nM (Table 3). Modification of the methyl group in the sulfonyl group of mCMV280 to an ethyl group (mCMY265) was well tolerated. Despite this, further modification to an isopropyl group (mCMY266) led to a significant decrease in activity, with 100% mortality dropping to 26% at 200 nM.

**Table 3:**
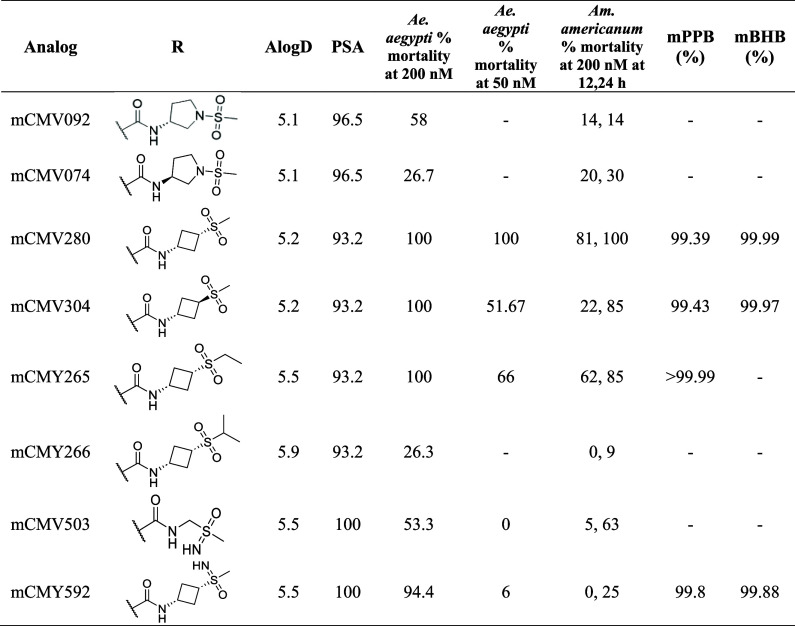
Profiling Data for the mCMV280 SAR Series^a^

a PSA = polar surface area, mPPB = mouse PPB, and mBHB = mouse BHB.

### Sulfoximide Derivatives of the Amide Tail

Sulfoximide derivatives were explored because of their increased PSA, reduced logD, and additional HBD. Among these, a ring open sulfoximide derivative (mCMV503) demonstrated moderate potency, achieving 53% mortality at 200 nM in mosquitoes. Importantly, mCMY592, which replaced the sulfonyl group of mCMV280 with sulfoximide, maintained high toxicity against mosquitoes but was 4x less toxic against ticks compared to mCMV280 (LC_50_ = 50 nM vs 14 nM). Based on good toxicity and novelty, mCMV280 and mCMY592 were both dosed in oral mouse PK studies with brain exposure measured over 24 h. Both compounds had substantially reduced brain exposure compared to fluralaner, with a B/P of 0.052 for mCMY592 and 0.12 for mCMV280 versus ∼1 for fluralaner. Considering the PPB and BHB, mCMV280 had a superior *K*_p,uu_ to mCMY592 (0.0015 vs 0.031). Based on the ectoparasiticidal activity, improved B/P, and *K*_p,uu_, mCMV280 was selected as the lead compound.

Mix and match SAR analogs were synthesized with the mCMV280 sulfonyl group and other modifications on the rest of the molecule from previous SAR explorations. As expected, most combinations were tolerated. Converting the internal aryl ring to a pyridine ring maintained the toxicity (Table 4, mCMX946). A CF_3_ group substitution on the internal aryl ring resulted in similar toxicity to mosquitoes when compared to mCMV280 but was less toxic to ticks at 24 h (87%). To reduce brain exposure even further, as seen with mCMN879 (Table 2), a cyano group was incorporated on the western aryl ring to give compound mCMX979. Unfortunately, this resulted in a decrease in the toxicity to mosquitoes.

**Table 4:**
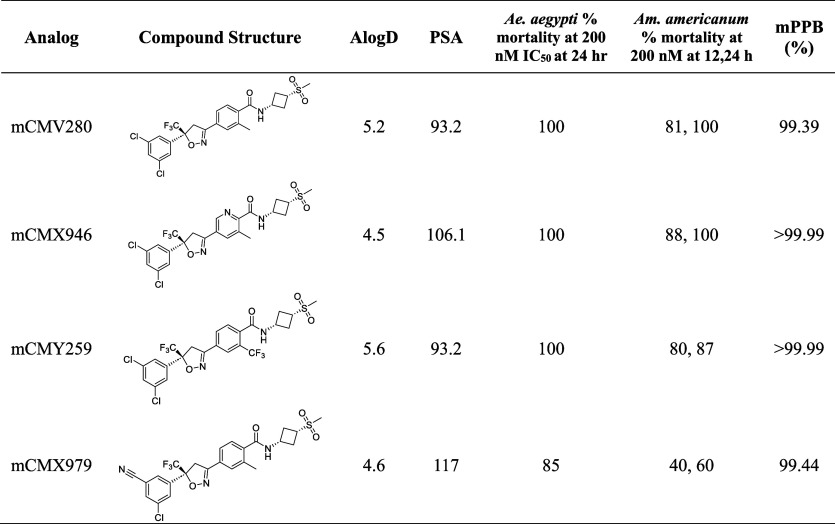
Profiling Data for the mCMV280 SAR Series^a^

a PSA = polar surface area and mPPB = mouse PPB.

The lead compound, mCMV280, was scaled up for PK studies across species (scheme and crystal X-ray diffraction shown in Supporting Information) and tested in in vitro safety studies. LC_50_ values were determined for mCMV280 in *Ae. aegypti*, *An. stephensi,* and *Am. americanum* which showed excellent concentration-dependent toxicity across all species. In *An. stephensi*, the LC_50_ for mCMV280 was comparable to that for fluralaner (31 vs 39 nM), but the toxicity of mCMV280 was 3x greater in ticks with an LC_50_ value of 54 nM (95% CI: 35–79 nM, Hillslope: 1.3, *R* ^2^: 0.98) when compared to fluralaner (LC_50_: 170 nM, 95% CI: 78–467 nM, Hillslope: 1.0, *R* ^2^: 0.91) at 12 h. However, in *Ae. aegypti*, the toxicity of mCMV280 was not significantly different from that of fluralaner with LC_50_ values of 27 nM (95% CI: 24–31 nM, Hillslope: 4.7, *R* ^2^: 0.88) for mCMV280 and 29 nM (95% CI: 28–31 nM, Hillslope: 4.7, *R* ^2^: 0.96) for fluralaner (Table 5).

**Table 5:** Comparative In Vitro and In Vivo Performance of Lead mCMV280 vs. Fluralaner^a^

	mCMV280	fluralaner
**In Vivo Toxicity-SMFA**		
*Ae. aegypti* LC_50_ at 24 h (nM)	27 (24–31, 4.7, 0.88)	29 (28–31, 4.7, 0.96)
(95% CI, Hillslope, *R* ^2^)		
*An. stephensi* LC_50_ at 24 h (nM)	31	39
(95% CI, Hillslope, *R* ^2^)		
*Am. americanum* LC_50_ at 12, 24 h (nM)	54 (35–79, 1.3, 0.98)	170 (78–467, 1.0, 0.91)
(95% CI, Hillslope, *R* ^2^)	14 (12–16, 1.4, 0.99)	48 (20–90, 1.1, 0.95)
**ADME Properties**		
CL_hep_, (μL/min/10^6^)	29.4 (ms), 3.73 (r), 4.37 (d), 4.0 (cy), 3.72 (h)	26.6 (ms), 11.3 (r),12.2 (d) 28.0 (cy), 4.22 (h)
MDCK MDR1 P_app_ A-B/B-A (10^–6^ cm/s)	12.1/15.4	6.12/3.95
PPB (%)	99.39 (ms), 99.86 (r), 99.89 (d), 99.95 (cy), 99.50 (h)	98.9 (ms), > 99.99 (r), 99.16 (d), > 99.99 (cy), > 99.99 (h)
BHB (%)	99.99 (ms)	99.98 (ms)
**Pharmacokinetic Parameters Following Single Mouse PO Dose**		
brain/plasma exposure at *C*_max_ (ng/mL)	1005/8281	4883/5164
B/P, *K*_p,uu_ at *C*_max,brain_	0.12, 0.0015	0.95, 0.017
**Target Selectivity and Tox Profiling**		
hERG channel IC_50_	11.6 μM (hERG), > 10 μM (NaV1.5, CaV1.2)	5.8 μM (hERG)
CYP inhibition IC_50_ (1A2, 2C19, 2C9, 2D6, 3A4)	2C9 = 34.1 μM, 2C19 = 49.5 μM, 1A2, 2D6, 3A4> 50 μM	2C9 = 11.6 μM, 2C19 = 12.7 μM, 2D6 = 17.5 μM, 1A2, 3A4 > 50 μM
Ames and MNT assay	negative	ND

a SMFA = standard membrane feeding assay, LC_50_ = 50% lethal concentration, ms = mouse, r = rat, d = dog, cy = cynomolgus monkey, Cl_hep_ = hepatic clearance, MDCK MDR1 = Madin–Darby canine kidney Multidrug Resistance 1 permeability assay, P_app_ = apparent permeability, A-B = apical-to-basolateral direction, B-A = basolateral-to-apical direction, PPB = plasma protein binding, BHB = brain homogenate binding, PO = per os (oral), *C*_max_ = maximum plasma concentration, B/*P* = brain-to-plasma ratio, *K*_p,uu_ = unbound brain-to-blood concentration ratio, *C*_max,brain_ = maximum concentration in the brain, hERG = human ether-a-go-go-related gene, and IC_50_ = half-maximal inhibitory concentration.

There were no major off-target concerns identified for mCMV280 in a cardiac panel screen (Nav1.5, Cav1.2, and hERG) with all IC_50_ values greater than 10 μM, which is an improvement from fluralaner that inhibits hERG with an IC_50_ of 5.8 μM (Table 5). The potential for genotoxicity and mutagenicity was evaluated in the MNT and Ames assays, respectively, where mCMV280 showed no safety concern with negative outputs for both. Assays measuring direct and time-dependent inhibition of CYP1A2, CYP2C9, CYP2C19, CYP2D6, and CYP3A4 in human microsomes showed minimal inhibition with all isoforms having an IC_50_ of ∼50 μM or greater except CYP2C9, where the IC_50_ was 34.1 μM (Table 5). The CYP3A4 IC_50_ remained >50 μM in the time-dependent inhibition study. Comparatively, fluralaner inhibition studies across CYP isoforms identified 3 with IC_50_ < 20 μM (CYP2C9, CYP2C19, and CYP2D6).

Oral and IV PK studies were conducted for mCMV280 in the mouse, rat, dog, and cyno monkeys. Plasma samples in PK studies in higher species were collected up to 1 month to provide accurate measurement of half-life and AUC_inf_. Figure 5 shows a summary of the PK data when mCMV280 was dosed via IV (dose 0.3–1 mg/kg, solution) and orally (1–5 mg/kg) as an aqueous suspension. mCMV280 showed very low clearance across species (<1 mL/min/kg, Figure 5), likely due to high PPB and good metabolic stability. As expected, the oral half-life for mCMV280 and fluralaner when dosed orally in dogs is long (250 vs 215 h), suggesting that mCMV280 would need infrequent dosing in drug-based vector control campaigns. Both compounds had shorter half-lives in cyno monkeys (103 and 90.5 h). mCMV280 showed moderate oral bioavailability across species, ranging from 20% in rats to 43% in cyno monkeys. In the dog PK study, with a single dose of 1 mg/kg, the concentration of mCMV280 in plasma was sufficient to cover the mosquito LC_90_ for up to 1 month. The accumulated in vitro and in vivo data were used to project the human PK profile and obtain an estimated human equivalent dose. For the TPP of an oral, once a month compound, the estimated human efficacious dose was 30 mg/kg. As expected, the human clearance is predicted to be low (0.05 mL/min/kg), with moderate oral bioavailability (31%). This low expected dose and infrequent dosing regime is ideal for the intended use where cost of goods and convenience is crucial for implementation.

**Figure 5:**
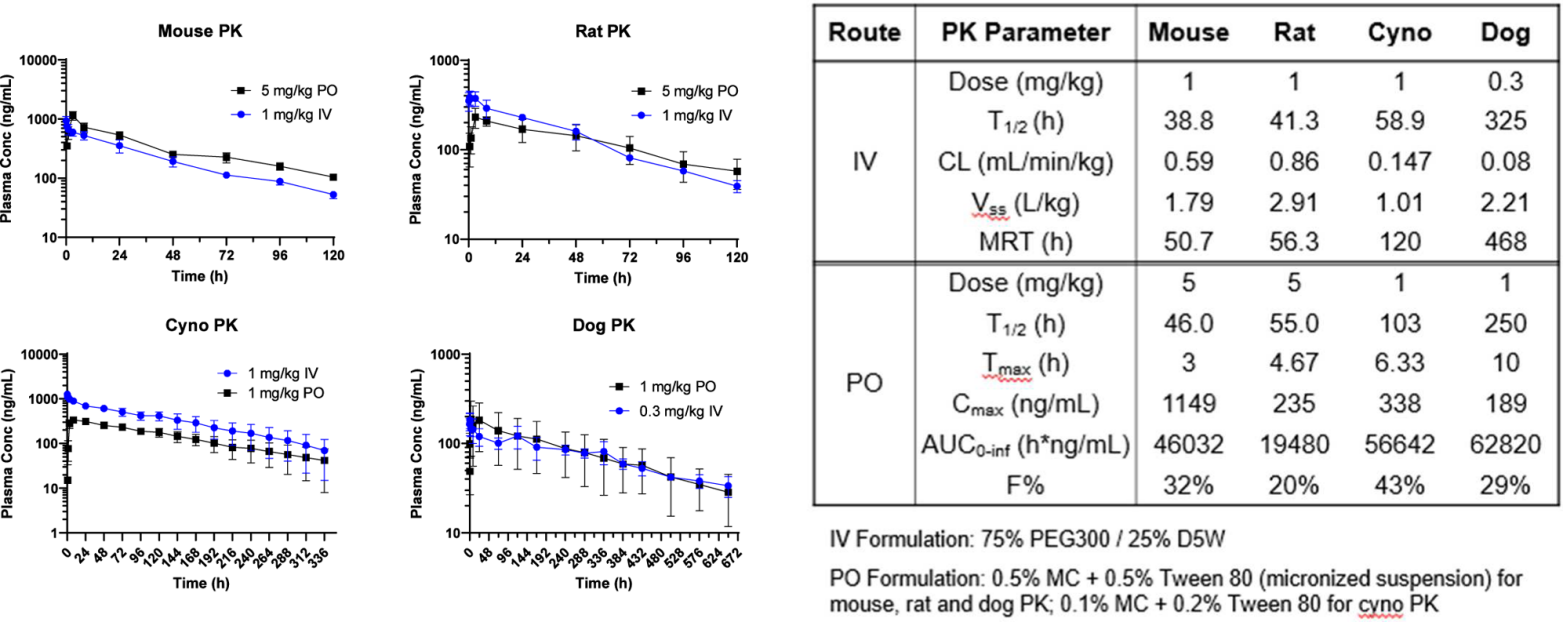
Pharmacokinetic summary of mCMV280 in the mouse, rat, dog, and cyno shows low clearance across species with a long half-life and moderate oral bioavailability. IV = intravenous, PO = per os (oral), *T*_1/2_ = half-life, CL = clearance, V_ss_ = volume of distribution at the steady state, MRT = mean residence time, *T*_max_ = time to maximum plasma concentration, *C*_max_ = maximum plasma concentration, AUC_0‑inf_ = area under the curve from time zero to infinity, and *F* = bioavailability.

## Conclusion

Despite global efforts, malaria remains one of the main infectious diseases to cause deaths worldwide, and Lyme disease is the leading vector-borne disease in the United States with an estimated >400,000 cases per year. A multifaceted approach is needed to reach the goals of malaria reduction^28,29^ and to reduce human morbidity from TBDs. This study describes the discovery of novel isoxazoline ectoparasitides that are equitoxic to commercialized ectoparasitides but with significantly reduced mammalian brain exposure and no observable off-target safety concerns in mammalian model systems. These novel compounds were designed to mitigate known CNS events caused by isoxazolines to enable a sufficient safety window for human dosing. Chemical modifications of the bis amide motif of fluralaner to increase the PSA lead to the desired profile of mCMV280 with reduced brain exposure and good toxicity against ticks and mosquitos. This program seeks to add an additional tool in the fight against malaria through oral dosing of the ectoparasiticide to the human population in endemic countries. This method stops the transmission cycle through reduction of the vector population, targets mosquitoes both indoors and outdoors, and has been projected to dramatically reduce malaria transmission.^22^ Additionally, lead mCMV280 shows promise as a prophylactic agent against most tick-vectored pathogens with the potential to kill the tick before transmission of most pathogenic agents.^30,31^ By optimizing the physicochemical properties of commercialized isoxazoline ectoparasitides, we imparted novelty into the chemical series and improved the safety profile of this class while maintaining high toxicity to arthropods. Considering this, mCMV280 represents an effective new tool in the fight against mosquito- and tick-vectored pathogens that cause human disease, as well as arthropod-vectored pathogens in livestock and companion animals.

## Materials and Methods

### Chemistry

Reagents and solvents were commercially obtained and used without further purification. Anhydrous solvents were used, and reactions were performed under an atmosphere of argon or nitrogen. Crude products were purified by silica gel chromatography or C18 reverse-phase columns. ^1^H NMR spectra were obtained on Bruker AV400 or AV500 instruments. Chemical shifts (δ) are expressed in parts per million (ppm), and coupling constants (*J*) in Hertz (Hz). LCMS was obtained on a Waters Acuity Ultra Performance LC system.

The commercialized isoxazolines fluralaner, lotilaner, sarolaner, and afoxolaner were obtained from Calibr-Skaggs at Scripps Research (La Jolla, CA, USA), where the compounds were either purchased or synthesized according to a literature procedure. Analogs of the compound fluralaner were synthesized at Calibr-Skaggs according to one of the three general schemes outlined in the Supporting Information. All compounds profiled in the paper have the (*S*) enantiomeric form of the isooxazoline ring with single-crystal X-ray diffraction for mCMV280 described in Supporting Information. The purity of all test compounds was determined to be ≥ 95% by ^1^H NMR and UPLC.

Scheme 1 is a representative scheme for the synthesis of fluralaner analogs. Boronic acids of the desired aryl were purchased or synthesized using standard conditions and then coupled with the vinyl halide using Suzuki conditions. **2** then gets reacted with key intermediate **B** and undergoes a 3 + 2 reaction to give the isoxazoline ring. Hydrolysis of the methyl ester, followed by amide coupling with the typical amine, gave the final compounds as racemic mixtures. These compounds were then separated by chiral separation to give single enantiomers, which were then tested in the SMFA to determine activity and distinguish between enantiomers (5*S* enantiomers are the active compounds).

#### 3-Chloro-5-(3,3,3-trifluoroprop-1-en-2-yl)­benzonitrile (**2**)

To a solution of (3-chloro-5-cyanophenyl)­boronic acid **1** (2.00 g, 11 mmol) in THF/H_2_O (2:1, 15 mL) was added Cs_2_CO_3_ (7.2 g, 22 mmol), and the mixture was degassed with N_2_ for 30 min. 2-Bromo-3,3,3-trifluoroprop-1-ene (2.1 g, 12 mmol) and PdCl_2_(PPh_3_)_2_ (0.15 g, 0.20 mmol) were added at room temperature, and the reaction mixture was stirred at 85 °C for 12 h. The reaction mixture was quenched with H_2_O (50 mL) and extracted with EtOAc (3 × 30 mL). The combined organic layers were dried over Na_2_SO_4_ and concentrated in vacuo to afford the crude material, which was purified by column chromatography (100% Hexanes) to yield **2** (2.3 g, 90%).

#### Methyl 4-(5-(3-chloro-5-cyanophenyl)-5-(trifluoromethyl)-4,5-dihydroisoxazol-3-yl)-2-methylbenzoate (**3**)

To a solution of methyl 4-((hydroxyimino)­methyl)-2-methylbenzoate **Inter**-**B** (1.18 g, 6 mmol) in DCM (35 mL) were added **2** (1.42 g, 6 mmol) and NaOCl (21.3 mL, 15 (v)) at room temperature, and the reaction mixture was stirred for 12 h. The reaction mixture was quenched with H_2_O (50 mL) and extracted with DCM (3 × 30 mL). The combined organic layers were dried in Na_2_SO_4_ and concentrated in vacuo. The crude material was purified by column chromatography (4% EtOAc in Hexanes) to afford **3** (1.7 g, 66%).

#### 4-(5-(3-Chloro-5-cyanophenyl)-5-(trifluoromethyl)-4,5-dihydroisoxazol-3-yl)-2-methylbenzoic Acid (**4**)

To a solution of **3** (0.20 g, 0.47 mmol) in MeOH (5 mL) was added a solution of LiOH (60 mg, 1.4 mmol) in H_2_O (0.5 mL) at room temperature, and the reaction mixture was stirred for 12 h. After completion, the mixture was concentrated under reduced pressure and diluted with H_2_O (5 mL), and the pH was adjusted to 2 with 1 M HCl. The product was extracted with DCM (3 × 20 mL), and the combined organic layers were dried over Na_2_SO_4_ and concentrated in vacuo to afford crude **4** (0.199 g, quantitative yield).

#### 4-(5-(3-Chloro-5-cyanophenyl)-5-(trifluoromethyl)-4,5-dihydroisoxazol-3-yl)-2-methyl-*N*-(2-oxo-2-((2,2,2-trifluoroethyl)­amino)­ethyl)­benzamide (**5**)

To a solution of **4** (0.18 g, 0.44 mmol) in DCM (5 mL) were added 2-amino-*N*-(2,2,2-trifluoroethyl)­acetamide (0.127 g, 0.66 mmol) and DMAP (5 mg, 0.04 mmol) at room temperature. EDC·HCl (126 mg, 0.66 mmol) in DCM (1 mL) was added to the stirred solution at room temperature, and the reaction mixture was stirred for 4 h. The reaction was quenched with H_2_O (10 mL) and extracted with DCM (3 × 5 mL). The combined organic layers were dried over Na_2_SO_4_ and concentrated in vacuo. The crude material was purified by preparative HPLC to afford **5** as a racemic mixture (29 mg, 12%). Both isomers were subsequently separated by SFC to yield mCMN879 as the active (*S*) compound.

### *Aedes aegypti* and *Amblyomma americanum* Rearing

*Aedes aegypti* used for this study were reared in the lab at the University of Florida Emerging Pathogens Institute in Gainesville, FL. Mosquito eggs were placed in room temperature water with a meal broth containing 3:1 parts of liver powder to Brewer’s yeast. Larvae were hatched and held in an environmental chamber at 27 °C with 50–60% relative humidity and a 14:10 h light:dark photoperiod. Pupae were collected and placed in cages with cotton filled with a 10% sucrose solution until adult eclosion. Adult female *Ae. aegypti* aged 3–5 days were used in membrane feeding studies.

*Amblyomma americanum* adult males and females were maintained in a colony at the Oklahoma State University Tick Rearing Facility (Stillwater, OK, USA). Ticks were maintained at conditions similar to those of *Ae. aegypti* in the same environmental chamber with enclosures containing moist towels to prevent desiccation. Both male and female ticks were used in subsequent ingestion membrane feeding experiments. All ticks purchased were declared by the supplier to be pathogen-free.

### Mosquito Toxicity Assay

Commercialized isoxazolines were tested against *Ae. aegypti* to establish the LC_50_ values for each compound. Groups of starved (∼24 h) adult female *Ae. aegypti* (5 to 7 days old, n = 15, rep = 3) were placed in 12 oz paper cups with a mesh lid, and various concentrations of each isoxazoline were fed in a blood meal through the mesh lid. Each blood meal was offered via a Hemotek membrane feeding system (Hemotek Ltd., Blackburn, UK) with parafilm stretched over the feeding apparatus to mimic skin. The groups of mosquitoes were allowed to feed ad libitum for 30 min before blood supply removal and were maintained in the environmental chamber until a 24 h mortality check. Mortality was assessed as the inability to return to the dorsal side up after prodding with a needle or lack of any movement. Concentration–response curves (CRCs) were generated for each isoxazoline using GraphPad Prism 10, and a nonlinear regression (variable-slope 4 parameters) was used to calculate LC_50_ values.

Further, over 100 analogs of the parent compound fluralaner were screened against cohorts of *Ae. aegypti* via the same blood-feeding procedure as described previously. Three replications of each compound at 200 nM were performed with the same description of mosquitoes as described above, and mortality was assessed 24 h post feeding. The % mortality from each replication was averaged, and any compound that did not elicit ≥ 80% mortality was not considered for future testing.

### Tick Ingestion Assay

Compounds rendering ≥ 80% mortality 24 h post feeding to mosquitoes were tested for activity against *Am. americanum* ticks via artificial blood meal feedings following previously described methods.^27,32^ Each blood meal was prepared with 10 μM adenosine triphosphate (ATP) as a feeding stimulant, 10 μM gentamicin sulfate for bacterial prevention, and 200 μM rhodamine B as a fluorescent indicator of feeding as previously described.^27,33,34^ Compounds were incorporated into a blood meal at 200 nM, and mortality was assessed every 12 h for 72 h. Compounds that yielded ≥ 50% mortality at this discriminatory concentration after 12 h of feeding were studied at 50 nM and 10 nM, and CRCs were constructed for compounds that resulted in >50% mortality at 50 nM. CRCs employed 7 concentrations ranging from 10 nM to 200 nM with 15 individual ticks per concentration. LC_50_ values were calculated from the CRCs through nonlinear regression (variable-slope 4 parameters) analysis in GraphPad Prism 10.

### In Vivo PK–PD Studies

Male Swiss CD1 mice were randomized into 5 groups of 2 mice each. Mice were dosed by oral gavage with vehicle (0.1% methylcellulose/0.2% Tween 80) or vehicle supplemented with 1, 4, 16, or 64 mg/kg of test compound kCEB596 (*S*-afoxolaner) of kCMF285 (*S*-fluralaner). 30 μL of blood samples (30 μL) were collected at 0, 1, 4, 24, 48, 96, 120, or 168 h after dosing as dried blood spots using Whatman DMPK-B cards. For PK analyses, samples were submitted to TC Life Sciences (Kolkata, India) where compounds were extracted using cold methylcyanide:H_2_O (80:20) and quantified by LC–MS against a standard curve diluted from a 10 mM stock solution in DMSO. *An. stephensi* female mosquitoes were allowed to feed on the shaved skin of the mice at 48, 120, 168, 216, 192, or 360 h post dosing, and mosquito mortality was recorded 48 h after feeding.

### In Vivo PK Studies

Detailed methods for PK determinates in rodents, dogs, and monkeys as DMPK studies are provided in the Supporting Information.

## Supplementary Material





## Notes

Additional synthetic procedures and characterization data including NMR spectra, DMPK assay, and in vitro assay information (PDF)
Molecular strings (CSV)

## Abbreviations used

**A**-B: apical-to-basolateral direction
B-A: basolateral-to-apical direction
BHB: brain homogenate binding
B/P: brain-to-plasma ratio
*C*_max_: maximum plasma concentration
*C*_max,brain_: maximum concentration in the brain
CNS: central nervous system
Cl_hep_: hepatic clearance
d: dog
hERG: human ether-a-go-go-related gene
IC_50_: half-maximal inhibitory concentration
*K*_p,uu_: unbound brain-to-blood concentration ratio
LC_50_: 50% lethal concentration
mBHB: mouse brain homogenate binding
MDCK MDR1: Madin–Darby canine kidney Multidrug Resistance 1 permeability assay
mPPB: mouse plasma protein binding
ms: mouse
MW: molecular weight
**r**: rat
*P*_app_: apparent permeability
PO: per os (oral)
PSA: polar surface area
PPB: plasma protein binding
SAR: structure activity relationship
SMFA: standard membrane feeding assay
*y*: cynomolgus monkey
