# Supplementary material for: Discovery of mCMV280: An Oral Ectoparasiticide in the Isoxazoline Class with Reduced Mammalian Brain Exposure
Source: J Med Chem. 2026 Mar 3;69(5):6199–209. doi: 10.1021/acs.jmedchem.5c03776 (PMC12990118; doi:10.1021/acs.jmedchem.5c03776)
Supplement: Supplementary file 1 [file jm5c03776_si_001.pdf]

Supporting Information
for

Discovery of mCMV280: An oral ectoparasiticide in the isoxazoline class with reduced mammalian brain exposure

Sarah E. McComic[1*], Katy B. Wilson[2*], Zhilin Li[1], Jeffrey Chen[2], Frank Weiss[2], Lirui Song[2], Wrickban Mazumdar[2], Curt A. Dvorak[2], Jason Brittain[2], Kyoung-Jin Lee[2], Shuangwei Li[2], Sean B. Joseph[2], Case W. McNamara[2], Martijn W. Vos[3], Avinash Sheshachalam[3], Alex Inácio[4], Koen J. Dechering[3], Arnab K. Chatterjee[2], Daniel R. Swale[1#]

* designates co-first authors. SEM and KBW contributed equally to this work.

[1]Department of Entomology and Nematology, Emerging Pathogens Institute, University of Florida, Gainesville, FL 32610, USA

[2]Calibr-Skaggs Institute for Innovative Medicines at Scripps Research, La Jolla, CA, 92037, USA

[3]TropIQ Health Sciences, Transistorweg 5, 6534 AT Nijmegen, The Netherlands

[4]QM Diagnostics, Transistorweg 5, 6534 AT Nijmegen, The Netherlands

#Contributing Author:
Daniel R. Swale, Ph.D.
Emerging Pathogens Institute
Department of Entomology and Nematology
University of Florida
Gainesville, FL 32610, USA
dswale@ufl.edu

Table of Contents

1. Experimental Procedures for Compounds……………………………………..…………. .S3
2. Characterization data of all compounds…………………………………………….…… .S13
3. DMPK Assays……………………………………………………………………….….. S19
4. Additional Figures/Tables……………………………………………………………….S21
5. Characterization and Spectral Data of mCMV280…………...…………………………S22
6. Single Crystal Determination of mCMV280……………………………………………S28

1. Experimental Procedures for Compounds

Materials and Methods

Reagents and solvents were commercially obtained and used without further purification. Anhydrous solvents were used and reactions performed under an argon atmosphere. Crude products were purified on silica gel chromatography or C18 reverse columns. ^{1}H NMR spectra were obtained on Bruker Ultrashield 400, 500, or 600 MHz. Shifts are expressed in ppm (parts per million) and coupling constants (J) in Hertz (Hz). LC-MS was obtained on Waters Acquity Ultra Performance LC. The purity of all test compounds was determined to be ≥95% by ^{1}H-NMR and UPLC/LCMS.

Known isoxazolines were purchased or synthesized according to literature procedures.
All other compounds were synthesized according to general route A, B, or C, depending on the compound. Compounds that were made as a racemic mixture were separated by chiral SFC, then each enantiomer was tested in the SMFA. The more potent enantiomer was assigned as the active (5*S*) enantiomer. All compounds profiled in the paper have the (*S*) enantiomeric form of the isooxazoline ring. The er (enantiomeric ratio) for the lead mCMV280 after SFC separation was >99:1.

Synthesis of Inter-A

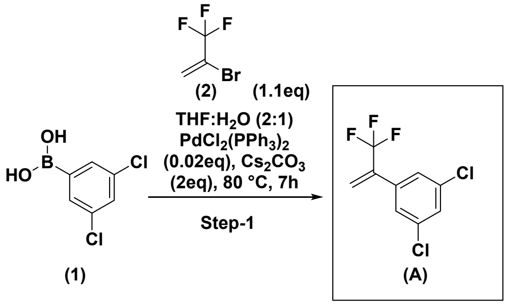

Scheme S1. Synthetic route used for synthesis of aryl alkenes

Step 1. To a solution of inter-1 (5.00 g, 26.3 mmol) in tetrahydrofuran/water (2:1, 50 mL) was added cesium carbonate (17.0 g, 52.6 mmol), and the mixture was degassed with nitrogen for 30 min. Inter-2 (5.03 g, 28.9 mmol) and palladium(II) chloride bis(triphenylphosphine) [$PdCl_2(PPh_3)_2$, 0.37 g, 0.52 mmol] were added at room temperature, and the reaction mixture was stirred at 85 °C for 5 h. The reaction was quenched with water (100 mL), and the product was extracted with ethyl acetate (3 × 50 mL). The combined organic layers were dried over anhydrous sodium sulfate and concentrated under reduced pressure to afford the crude material, which was purified by column chromatography on silica gel. Elution with 100% hexane afforded Inter-A (4.60 g, 73% yield).

Synthesis of Inter-B

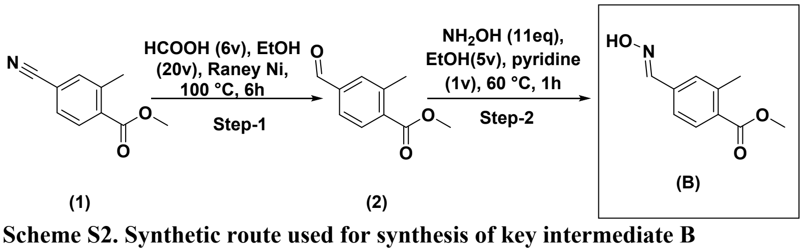

Scheme S2. Synthetic route used for synthesis of key intermediate B

Step 1. To a solution of inter-1 (6.00 g, 34.2 mmol) in ethanol (120 mL, 20 v) were added formic acid (36 mL, 6 v) and Raney nickel (12.0 g) under a nitrogen atmosphere, and the reaction mixture was stirred at 100°C for 6 h. The mixture was filtered through Celite, and the filtrate was washed with methanol and

concentrated under reduced pressure to afford the crude material which was purified by column chromatography on silica gel. Elution with 6% ethyl acetate in hexanes afforded inter-2 (5.00 g, 81.9% yield).

Step 2. To a solution of inter-2 (1.40 g, 7.80 mmol) in ethanol (7 mL) were added hydroxylamine hydrochloride ($NH_2OH \cdot HCl$, 5.90 g, 84.9 mmol) and pyridine (1.40 mL) at room temperature, and the reaction mixture was stirred at 60 °C for 1 h. The mixture was poured into ice water (50 mL) to obtain a solid, which was filtered and washed with water (50 mL). The solid was dissolved in dichloromethane (DCM, 50 mL), dried over anhydrous sodium sulfate, and concentrated under reduced pressure to afford pure inter-B (1.40 g, 79% yield).

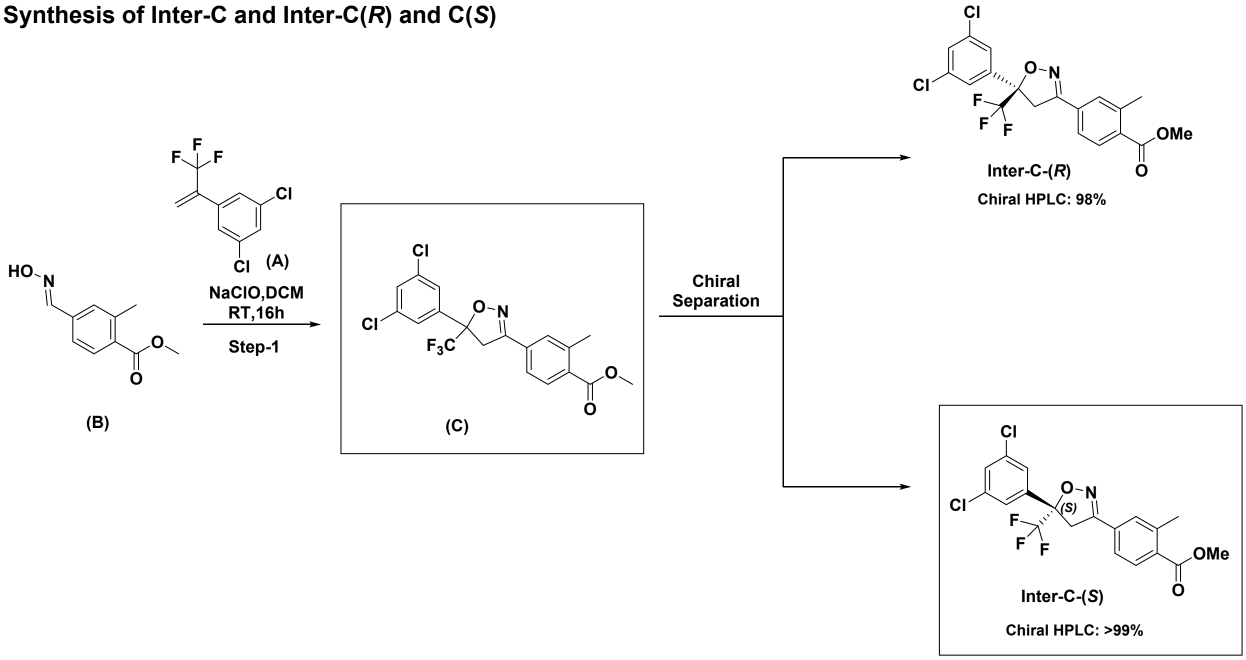

Scheme S3. Synthetic route used for amide SAR (single enantiomer)

Step 1. To a solution of inter-B (5.82 g, 30.0 mmol) in DCM (145 mL) were added inter-A (7.23 g, 30.0 mmol) and sodium hypochlorite (NaOCl, 87 mL, 15 v) dropwise at room temperature, and the reaction mixture was stirred for 12 h. The mixture was quenched with water (100 mL), and the product was extracted with DCM (3 × 50 mL). The combined organic layers were dried over anhydrous sodium sulfate and concentrated under reduced pressure to afford the crude material, which was purified by column chromatography on silica gel. Elution with 4% ethyl acetate in hexanes afforded inter-C (8.00 g, 61.4% yield). Chiral SFC was used to separate the (*R*)- and (*S*)-enantiomers of the ester intermediate, yielding Inter-C-(*R*) and Inter-C-(*S*), respectively. The (*S*)-enantiomer provided final compounds with the correct stereocenter for potency in the SMFA.

<u>General Route A:</u>

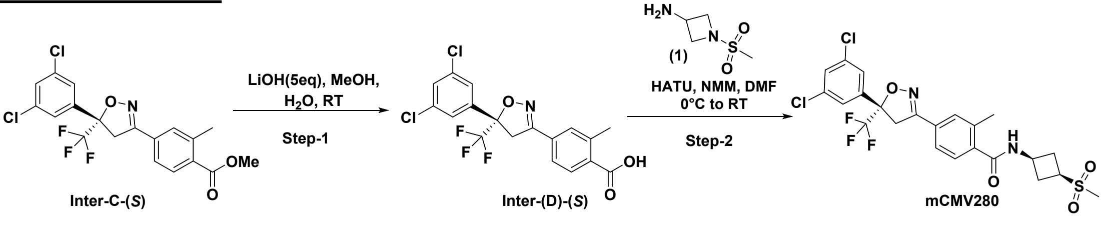

Scheme S4. Synthetic route used for amide SAR (single enantiomer)

General route A: The synthetic route to mCMV280 is described below in scheme one. Analogs with SAR on the amide were made using Inter-C-(*S*) under standard amide coupling conditions. The (*S*) enantiomeric form of the isoxazoline is known to be the active enantiomer, so this intermediate was used for all amide SAR described in Scheme 4.

Step 1. Inter-C-(*S*) (108 mg, 0.25 mmol, 5 equiv) was dissolved in 1 mL THF under an argon atmosphere. LiOH (30 mg, 1.25 mmol) was dissolved in H_2O (0.35 mL) and added to the solution with stirring. The mixture was heated to 60 °C until the reaction was complete (~2–3 h) or stirred at room temperature for extended periods. The reaction mixture was acidified to pH < 4 and extracted with EtOAc. The combined organic layers were dried over Na_2SO_4 and concentrated *in vacuo* to afford inter-D-(*S*) (101 mg, 0.241 mmol, 96% yield).

Step 2. Inter-D-(*S*) (24 mg, 0.057 mmol) and HATU (24 mg, 0.063 mmol, 1.1 equiv) were dissolved in DMF (1 mL) and cooled to 0 °C, followed by addition of amine 1 (13 mg, 0.068 mmol, 1.2 equiv) and N-methylmorpholine (NMM, 20.6 µL, 0.187 mmol, 3.3 equiv). The resulting solution was allowed to warm to room temperature and monitored by LCMS. Upon completion, the reaction was quenched with H_2O and extracted with EtOAc. The combined organic layers were washed with brine, dried over Na_2SO_4, and concentrated *in vacuo*. The crude residue was dissolved in minimal DMSO and purified by C18 column chromatography (MeCN/H_2O) to afford mCMV280 (15.7 mg, 0.029 mmol, 50% yield).

General Route B:

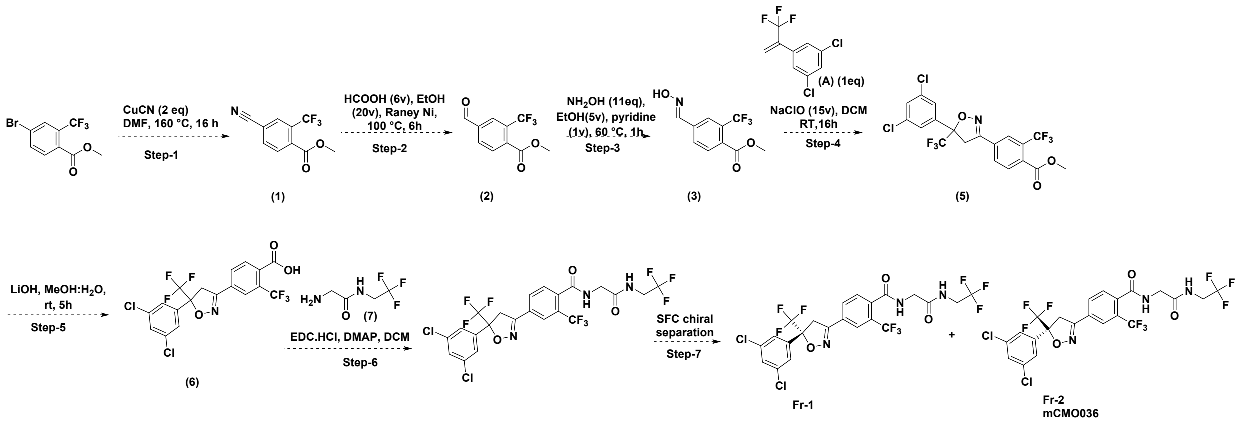

Scheme S5. General synthetic route used for internal aryl SAR

Internal aryl SAR analogs were synthesized following Scheme 5, reacting aryl halides under SNAr conditions to add a cyano group. Steps 2-7 follow the same procedure as described in the Schemes 1-4. The cyano group is reduced to an aldehyde. The aldehyde is then reacted with hydroxyl amine to give an oxime. Chlorination of the oxime can either be done in a separate step or in a one pot synthesis. Addition of the aryl alkene enables the 3 + 2 cyclization to form the isoxazoline ring racemically. Chiral SFC separation gives the two enantiomers which were tested in the SMFA to confirm activity and assign stereochemistry.

Step 1. The aryl bromide (1.04 g, 3.7 mmol, 1 equiv) and CuCN (663 mg, 7.4 mmol, 2 equiv) were dissolved in anhydrous DMF under an argon atmosphere and heated to 160 °C overnight. The mixture was diluted with DCM and H_2O, and the aqueous layer was extracted with DCM three times. The combined organic layers were washed with brine, dried over Na_2SO_4, and concentrated *in vacuo*. The crude residue was purified by flash silica gel column chromatography (hexanes/EtOAc) to afford compound 1 (532 mg, 60% yield).

General Route C:

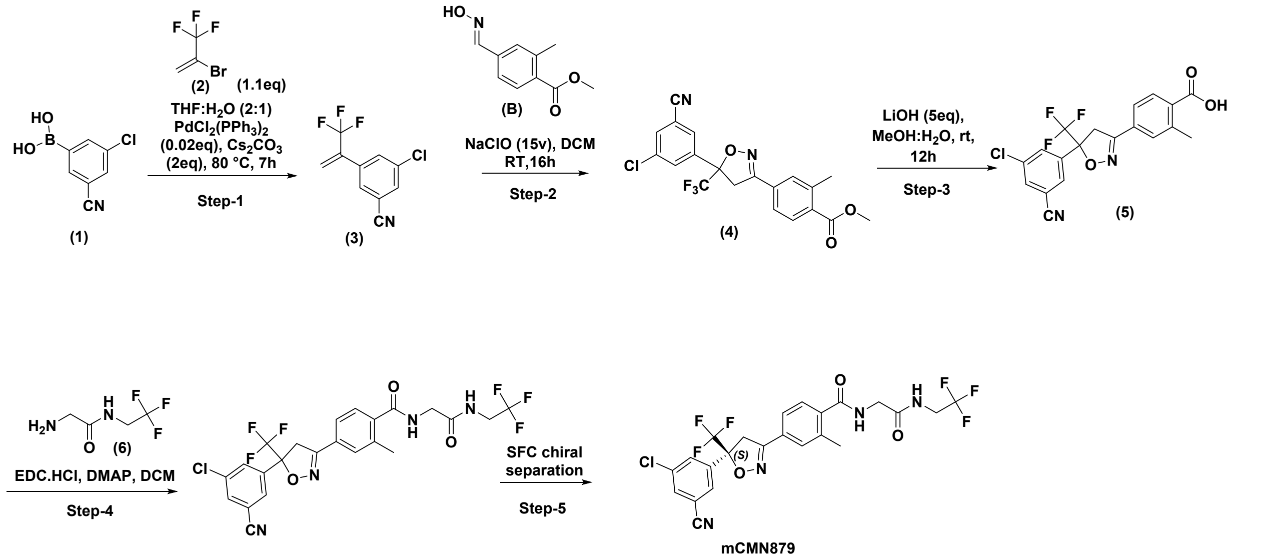

Scheme S6. Synthetic route used for western aryl SAR

Western aryl analogs were made according to Scheme 2. Boronic acids of the desired aryl were purchased or synthesized using standard conditions, then coupled with the appropriate vinyl halide using Suzuki conditions. Intermediate 3 then gets reacted with key intermediate B and undergoes a 3 +2 reaction to form the isoxazoline ring. Hydrolysis of the methyl ester, followed by amide coupling with the typical amine, gives final compounds as racemic mixtures. These compounds were then separated by SFC chiral separation to give single enantiomers, which were then tested in the SMFA to determine activity and distinguish between enantiomers (5*S* enantiomers are the active compounds).

Step 1. To a solution of inter-1 (2.00 g, 11 mmol) in THF/H_2O (2:1, 15 mL) was added Cs_2CO_3 (7.2 g, 22 mmol), and the mixture was degassed with N_2 for 30 min. Inter-2 (2.1 g, 12 mmol) and $PdCl_2(PPh_3)_2$ (0.15 g, 0.20 mmol) were added at room temperature, and the reaction mixture was stirred at 85 °C for 12 h. The reaction mixture was quenched with H_2O (50 mL) and extracted with EtOAc (3 × 30 mL). The combined organic layers were dried over Na_2SO_4 and concentrated *in vacuo* to afford crude material, which was purified by column chromatography on silic gel (100% hexanes) to yield inter-3 (2.3 g, 90%).
Step 2. To a solution of inter-B (1.18 g, 6 mmol) in DCM (35 mL) were added inter-3 (1.42 g, 6 mmol) and NaOCl (21.3 mL, 15 v) at room temperature, and the reaction mixture was stirred for 12 h. The reaction mixture was quenched with H_2O (50 mL) and extracted with DCM (3 × 30 mL). The combined organic layers were dried over Na_2SO_4 and concentrated *in vacuo*. The crude material was purified by column chromatography on silica gel (4% EtOAc in hexanes) to afford inter-4 (1.7 g, 66%).
Step 3. To a solution of inter-4 (0.20 g, 0.47 mmol) in MeOH (5 mL) was added a solution of LiOH (60 mg, 1.4 mmol) in H_2O (0.5 mL) at room temperature, and the reaction mixture was stirred for 12 h. After completion, the mixture was concentrated under reduced pressure and diluted with H_2O (5 mL), and the pH was adjusted to 2 with 1 M HCl. The product was extracted with DCM (3 × 20 mL), and the combined organic layers were dried over Na_2SO_4 and concentrated *in vacuo* to afford crude inter-5 (0.199 g, quantitative yield).
Step 4. To a solution of inter-5 (0.18 g, 0.44 mmol) in DCM (5 mL) were added inter-6 (0.127 g, 0.66 mmol) and DMAP (5 mg, 0.04 mmol) at room temperature. EDC·HCl (126 mg, 0.66 mmol) in DCM (1 mL) was added to the stirred solution at room temperature, and the reaction mixture was stirred for 4 h. The mixture was quenched with H_2O (10 mL) and extracted with DCM (3 × 5 mL). The combined organic layers were dried over Na_2SO_4 and concentrated *in vacuo*. The crude material was purified by preparative HPLC to afford mCMG229 racemic (29 mg, 12%). Both isomers were subsequently separated by SFC to yield mCMN879.

Synthesis of mCMV503

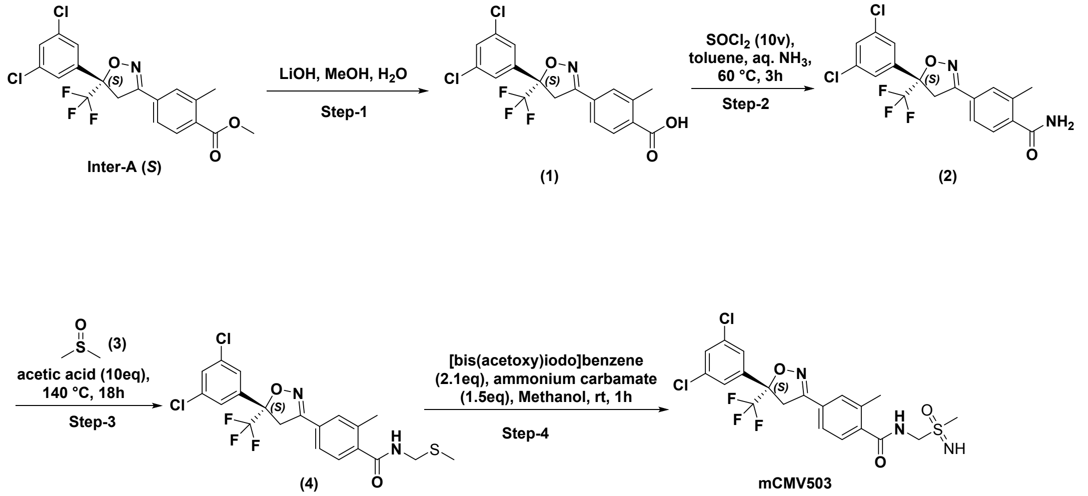

Scheme S7. Synthetic route for mCMV503

Step 2. To a solution of inter-1 (0.170 g, 0.40 mmol) in toluene (5 mL) were added DMF (0.1 mL) and thionyl chloride (0.17 mL, 10 v), and the reaction mixture was stirred at 60 °C for 1 h. Upon completion, the mixture was concentrated under vacuum, and the crude residue was diluted with toluene (2 mL). Aqueous NH_3 (5 mL, 30 v) was added at 0 °C, and the mixture was stirred at the same temperature for 15 min. The reaction mixture was poured into ice water (15 mL) and extracted with EtOAc (3 × 15 mL). The combined organic layers were dried over Na_2SO_4 and concentrated *in vacuo*. The crude material was purified by column chromatography on silica gel (1% MeOH in DCM) to afford inter-2 (0.16 g, 84%).
Step 3. To a solution of inter-2 (0.160 g, 0.30 mmol) in DMSO (5 mL) was added AcOH (0.230 g, 3.0 mmol) at room temperature, and the reaction mixture was stirred at 140 °C for 24 h. The reaction mixture was poured into ice water (10 mL) and extracted with EtOAc (3 × 15 mL). The combined organic layers were dried over Na_2SO_4 and concentrated in vacuo. The crude material was purified by column chromatography (15% EtOAc in hexanes) to afford Inter-3 (0.100 g, 55%).
Step 4. To a solution of inter-4 (0.10 g, 0.20 mmol) in MeOH (10 mL) were added ammonium carbamate (0.022 g, 0.35 mmol) and [bis(acetoxy)iodo]benzene (0.128 g, 0.42 mmol) at room temperature, and the reaction mixture was stirred for 2 h. Upon completion, the reaction mixture was concentrated, and the crude product was purified by preparative HPLC to afford mCMV503 (0.0198 g, 19%).

Synthesis of mCMV074

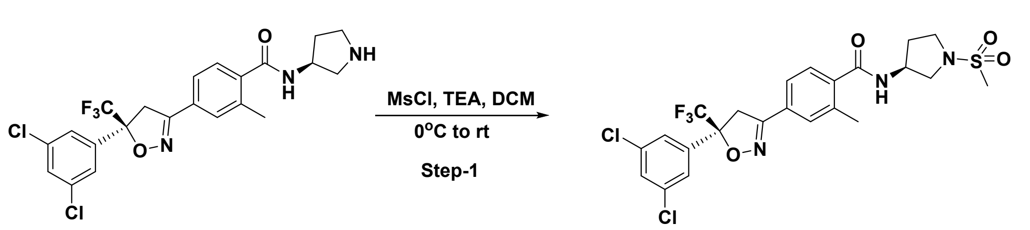

Scheme S8. Synthetic route for mCMV074

Step 1. To a stirred solution of 4-((S)-5-(3,5-dichlorophenyl)-5-(trifluoromethyl)-4,5-dihydroisoxazol-3-yl)-2-methyl-N-((S)-pyrrolidin-3-yl)benzamide (54 mg, 0.11 mmol, 1 equiv) and TEA (46 μL, 0.33 mmol, 3 equiv) in DCM (1 mL) was added methanesulfonyl chloride (20 μL, 0.22 mmol, 2 equiv) at 0 °C. The reaction mixture was stirred at room temperature for 2 h, and the progress was monitored by LCMS. Upon completion, the reaction mixture was poured into ice-cold water and extracted with EtOAc (3 × 50 mL). The combined organic layers were dried over anhydrous Na_2SO_4, filtered, and concentrated *in vacuo*. The

crude product was purified by flash column chromatography on silica gel (70% EtOAc/hexanes) to afford 4-((S)-5-(3,5-dichlorophenyl)-5-(trifluoromethyl)-4,5-dihydroisoxazol-3-yl)-2-methyl-N-((S)-1-(methylsulfonyl)pyrrolidin-3-yl)benzamide (22 mg, 35% yield).

Synthesis of mCMF883

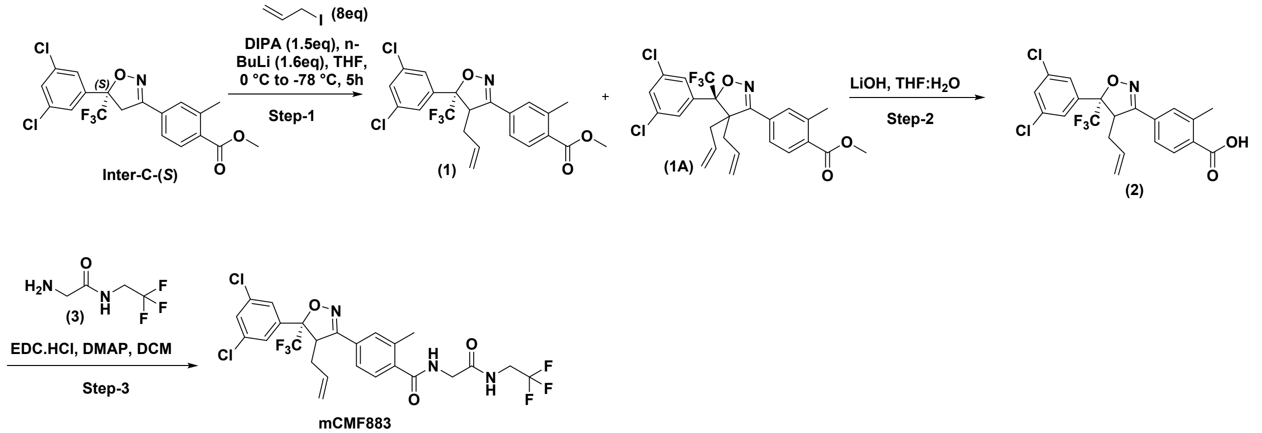

Scheme S9. Synthetic route for mCMF883

Step 1. To a stirred solution of DIPA (175 mg, 1.74 mmol) in THF (2 mL) was added n-BuLi (119 mg, 1.86 mmol) at 0 °C, and the mixture was stirred for 30 min. Inter-C-(S) (500 mg, 1.16 mmol) in THF (2 mL) was added at −78 °C, and the reaction mixture was stirred for 30 min. Upon consumption of inter-C-(S) and formation of a new polar spot, allyl iodide (1.55 g, 9.28 mmol) was added at −78 °C, and the mixture was stirred for 5 h at −78 °C. The reaction was quenched with H_2O (25 mL) and extracted with EtOAc (3 × 10 mL). The combined organic layers were dried over Na_2SO_4 and concentrated *in vacuo*. The crude material was purified by column chromatography (EtOAc/hexanes) to afford a mixture of inter-1 and inter-1A (70 mg, 12%). LCMS analysis indicated 62% inter-1 and 27% inter-1A.

Step 2. To a solution of inter-1 and 1A (340 mg, 0.722 mmol) in MeOH (6.8 mL) was added a solution of LiOH (151 mg, 3.61 mmol) in H_2O (0.68 mL) at room temperature, and the mixture was stirred at 6 °C for 4 h. Upon completion, the reaction mixture was concentrated under reduced pressure and diluted with H_2O (5 mL). The pH was adjusted to 3–4 using 1 M HCl, and the resulting solid was collected by filtration and washed with H_2O (25 mL) to afford inter-2 and 2A (280 mg, 85%). LCMS analysis indicated 59% inter-2 and 25% inter-2A.

Step 3. To a solution of inter-2 and 2A (200 mg, 0.438 mmol) in DCM (5 mL) were added inter-3 (126 mg, 0.656 mmol) and DMAP (5 mg, 0.044 mmol) at room temperature. EDC·HCl (126 mg, 0.656 mmol) in DCM (1 mL) was added to the stirred solution at room temperature, and the reaction mixture was stirred for 4 h. The mixture was quenched with H_2O (10 mL) and extracted with DCM (3 × 5 mL). The combined organic layers were dried over Na_2SO_4 and concentrated *in vacuo*. The crude product was purified by preparative HPLC to afford mCMF883 (12.2 mg, 4.7%).

Synthesis of mCMY265

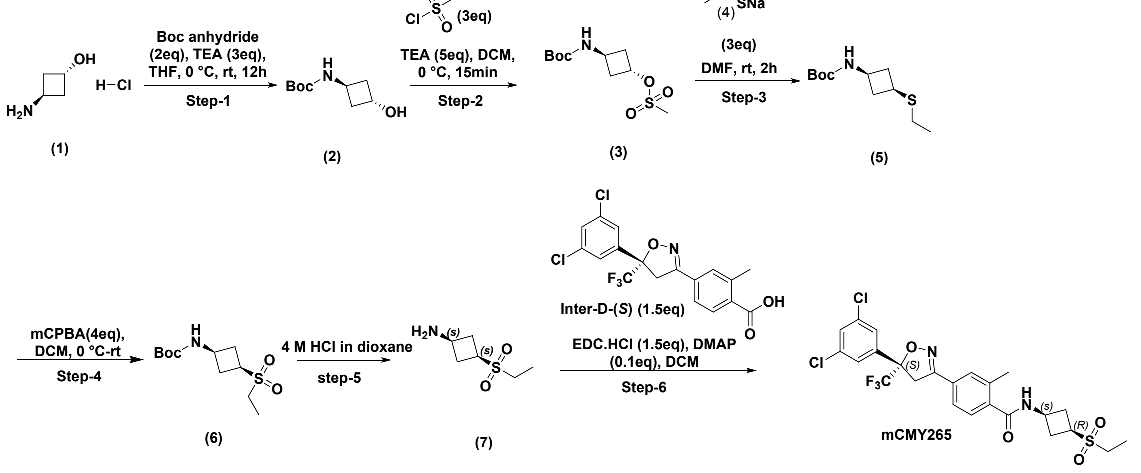

Scheme S10. Synthetic route for mCMY265

Step 1. To a solution of compound 1 (1.00 g, 8.13 mmol) in THF (10 mL) was added TEA (2.46 g, 24.39 mmol) at room temperature, and the mixture was stirred for 10 min. A solution of Boc anhydride (3.55 g, 16.26 mmol) in THF was then added dropwise at 0 °C, and the reaction mixture was stirred at room temperature for 16 h. The progress of the reaction was monitored by TLC. The reaction mixture was diluted with H_2O (40 mL) and extracted with EtOAc (3 × 40 mL). The combined organic layers were dried over Na_2SO_4 and concentrated *in vacuo*. The crude product was purified by column chromatography (20% EtOAc in hexanes) to afford the desired product (1.20 g, 79%).
Step 2. To a solution of compound 2 (1.00 g, 5.32 mmol) in DCM (10 mL) was added TEA (1.61 g, 15.95 mmol) at room temperature, and the mixture was stirred for 10 min. A solution of mesyl chloride in DCM was added dropwise at 0 °C, and the reaction mixture was stirred at 0 °C for 15 min. The progress of the reaction was monitored by TLC. The reaction mixture was diluted with H_2O (40 mL) and extracted with DCM (3 × 40 mL). The combined organic layers were dried over Na_2SO_4 and concentrated *in vacuo*. The crude product was purified by column chromatography on silica gel (20% EtOAc in hexanes) to afford the desired product (1.20 g, 85%).
Step 3. To a solution of compound 3 (0.40 g, 1.50 mmol) in DMF (10 mL) was added compound 4 (0.38 g, 4.50 mmol) at room temperature, and the reaction mixture was stirred for 4 h. The progress of the reaction was monitored by TLC. The reaction mixture was diluted with H_2O (20 mL) and extracted with EtOAc (3 × 20 mL). The combined organic layers were dried over Na_2SO_4 and concentrated in vacuo. The crude product was purified by column chromatography (5% EtOAc in Hexanes) to afford the desired product (0.170 g, 49%). LCMS: *m/z* = 232.15 $(M+1)^+$.
Step 4. To a solution of compound 5 (170 mg, 0.73 mmol) in DCM (10 mL) was added meta-chloroperbenzoic acid (m-CPBA, 500 mg, 2.94 mmol) at 0 °C. The reaction mixture was stirred at room temperature for 12 h, and the progress was monitored by TLC. Upon completion, the mixture was diluted with H_2O (20 mL) and extracted with DCM (3 × 15 mL). The combined organic layers were dried over anhydrous Na_2SO_4 and concentrated in vacuo. The crude product was purified by column chromatography on silica gel (15% EtOAc in hexanes) to afford the desired product as a solid (80 mg, 41%).
Step 5. To a solution of compound 6 (80 mg, 0.30 mmol) in dichloromethane (DCM, 2 mL), was added hydrogen chloride in dioxane (2 mL, 4M) dropwise at 0 °C. The reaction mixture was stirred at room temperature for 2 h. Progress of the reaction was monitored by TLC. After completion, the mixture was diluted with water (20 mL) and extracted with DCM (3 × 15 mL). The combined organic layers were separated, dried over anhydrous sodium sulfate, and concentrated under reduced pressure to afford the crude product (50 mg), which was carried forward to the next step without further purification. LC-MS (ESI): *m/z* = 164.05 $[M + H]^+$

Step 6. To a solution of compound 7 (50 mg, 0.30 mmol) in DCM (2 mL), 4-dimethylaminopyridine (DMAP, 3.7 mg, 0.030 mmol) and compound B (127 mg, 0.28 mmol) were added at room temperature and stirred for 5 min. Subsequently, N-(3-dimethylaminopropyl)-N′-ethylcarbodiimide hydrochloride (EDC·HCl, 88 mg, 0.46 mmol) was added, and the mixture was stirred for an additional 3 h at room temperature. Reaction progress was monitored by TLC. Upon completion, the mixture was diluted with water (10 mL) and extracted with DCM (3 × 10 mL). The combined organic layers were dried over anhydrous sodium sulfate and concentrated under reduced pressure. The crude product was purified by column chromatography (2.5% methanol in DCM) to yield mCMY265 (30 mg, 10% yield).

Synthesis of mCMY266

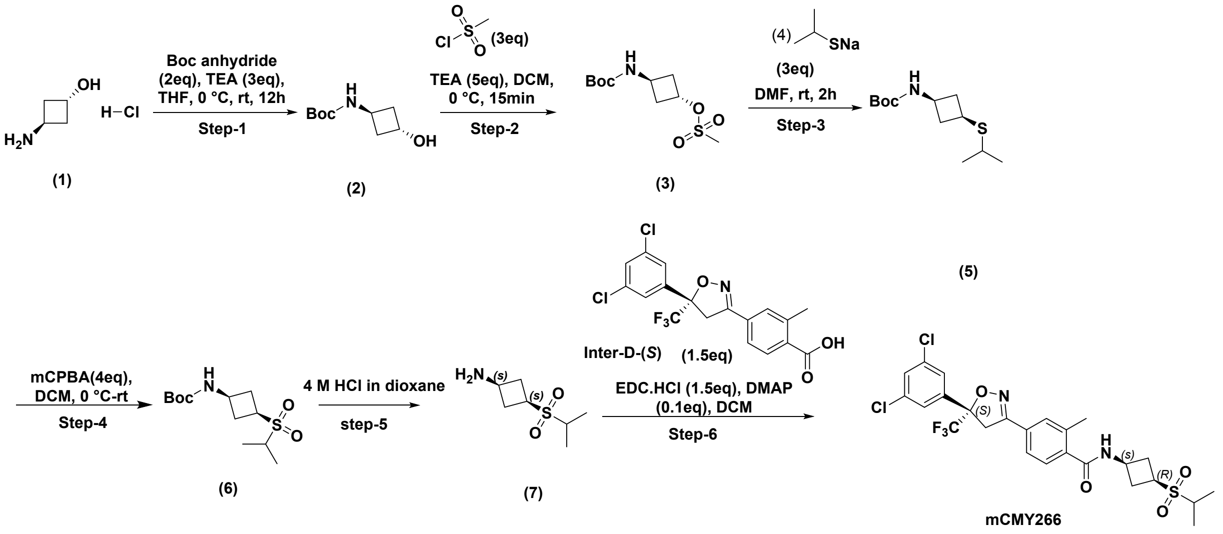

Scheme S11. Synthetic route for mCMY266

Step 1. To a solution of compound 1 (1.0 g, 8.13 mmol) in THF (10 mL) was added triethylamine (TEA, 2.46 g, 24.39 mmol) at room temperature and stirred for 10 min at the same temperature. A solution of Boc anhydride (3.55 g, 16.26 mmol) in THF was then added dropwise at 0 °C, and the mixture was stirred for 16 h at room temperature. Reaction progress was monitored by TLC. The mixture was diluted with H_2O (40 mL) and extracted with EtOAc (3 × 40 mL). The combined organic layers were dried over anhydrous sodium sulfate and concentrated under reduced pressure. The crude product was purified by column chromatography on silica gel using 20% ethyl acetate in hexane to afford the desired product (1.2 g, 79.2% yield). LC-MS (ESI): m/z = 215.16 $[M + H]^+$.

Step 2. To a solution of compound 2 (1.0 g, 5.32 mmol) in DCM (10 mL) was added TEA (1.61 g, 15.95 mmol) at room temperature and stirred for 10 min. A solution of methanesulfonyl chloride in DCM was then added at 0 °C, and the reaction was stirred for 15 min at the same temperature. TLC was used to monitor reaction progress. The mixture was diluted with saturated water (40 mL) and extracted with DCM (3 × 40 mL). The organic layer was dried over anhydrous sodium sulfate and concentrated under reduced pressure. The crude product was purified by column chromatography on silica gel using 20% ethyl acetate in hexane to yield the desired product (1.2 g, 84.7% yield).

Step 3. To a solution of compound 3 (400 mg, 1.50 mmol) in DMF (10 mL), compound 4 (443 mg, 4.50 mmol) was added at room temperature and stirred for 4 h. Reaction progress was monitored by TLC. The reaction mixture was diluted with water (20 mL) and extracted with ethyl acetate (3 × 20 mL). The combined organic layers were dried over anhydrous sodium sulfate and concentrated under reduced pressure. The crude product was purified by column chromatography on silica gel using 5% ethyl acetate in hexane to afford the desired product (120 mg, 32.4% yield). LC-MS (ESI): m/z = 246.15 $[M + H]^+$.

Step 4. To a solution of compound 5 (170 mg, 0.694 mmol) in DCM (10 mL) was added meta-chloroperbenzoic acid (mCPBA, 470 mg, 2.77 mmol) at 0 °C, and the reaction was stirred for 12 h at room temperature. TLC was used to monitor reaction progress. The reaction mixture was diluted with

water (20 mL) and extracted with DCM (3 × 15 mL). The combined organic layers were dried over anhydrous sodium sulfate and concentrated under reduced pressure. The crude product was purified by column chromatography on silica gel using 15% ethyl acetate in hexane to afford intermediate 6 (120 mg, 62.5% yield).

Step 5. To a solution of compound 6 (120 mg, 0.433 mmol) in DCM (2 mL) was added hydrogen chloride in dioxane (3 mL, 20% v/v) at 0 °C, and the reaction was stirred for 2 h at room temperature. TLC was used to monitor reaction progress. The reaction mixture was diluted with water (20 mL) and extracted with DCM (3 × 15 mL). The organic layer was dried over anhydrous sodium sulfate and concentrated under reduced pressure to afford the crude product (50 mg), which was carried forward to the next step without purification. LC-MS (ESI): *m/z* = 178.05 $[M + H]^+$.

Step 6. To a solution of compound 7 (50 mg, 0.28 mmol) in DCM (2 mL), 4-dimethylaminopyridine (DMAP, 3.4 mg, 0.028 mmol) and compound B (117 mg, 0.28 mmol) were added at room temperature and stirred for 5 min. N-(3-Dimethylaminopropyl)-N′-ethylcarbodiimide hydrochloride (EDC·HCl, 80 mg, 0.42 mmol) was then added, and the reaction mixture was stirred for an additional 3 h at room temperature. Reaction progress was monitored by TLC. The mixture was diluted with water (10 mL) and extracted with DCM (3 × 10 mL). The combined organic layers were dried over anhydrous sodium sulfate and concentrated under reduced pressure. The crude product was purified by column chromatography on silica gel using 2.5% methanol in DCM to afford mCMY266 (58 mg, 35.6% yield).

Synthesis of mCMY592

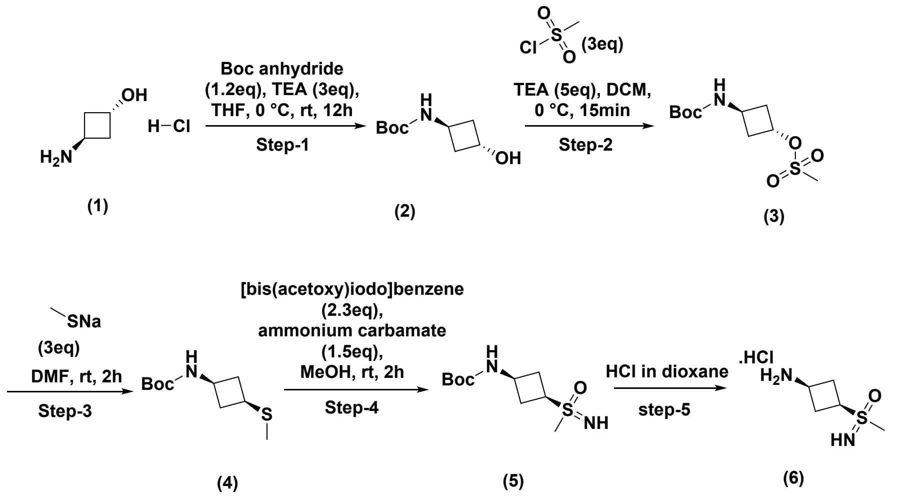

Scheme S12. Synthetic route for mCMY592

Step 1. To a solution of compound 1 (1.0 g, 8.13 mmol) in THF (10 mL) was added triethylamine (TEA, 2.46 g, 24.39 mmol) at room temperature and stirred for 10 min at the same temperature. A solution of Boc anhydride (3.55 g, 16.26 mmol) in THF was then added dropwise at 0 °C, and the mixture was stirred for 16 h at room temperature. Reaction progress was monitored by TLC. The mixture was diluted with water (40 mL) and extracted with ethyl acetate (3 × 40 mL). The combined organic layers were dried over anhydrous sodium sulfate and concentrated under reduced pressure. The crude product was purified by column chromatography using 20% ethyl acetate in hexane to afford the desired product (1.2 g, 79.2% yield). LC-MS (ESI): *m/z* = 215.16 $[M + H]^+$.

Step 2. To a solution of compound 2 (1.0 g, 5.32 mmol) in DCM (10 mL) was added triethylamine (TEA, 1.61 g, 15.9 mmol) at room temperature, and the mixture was stirred for 10 min at the same temperature. A solution of methanesulfonyl chloride (0.918 g, 8.00 mmol) in DCM (1 mL) was then added at 0 °C, and the reaction mixture was stirred for 15 min at that temperature. The reaction progress was monitored by TLC. The mixture was diluted with water (40 mL) and extracted with DCM (3 × 40 mL). The combined organic layers were dried over anhydrous sodium sulfate and concentrated under reduced pressure to afford the crude product, which was purified by column chromatography on silica gel using 20% ethyl acetate in hexane to yield the desired product (1.2 g, 84.7% yield).

Step 3. To a solution of compound 3 (0.400 g, 1.50 mmol) in DMF (10 mL) was added sodium methanethiolate (0.380 g, 4.50 mmol) at room temperature, and the reaction mixture was stirred for 4 h at the same temperature. The reaction progress was monitored by TLC. The mixture was diluted with water (20 mL) and extracted with ethyl acetate (3 × 20 mL). The combined organic layers were dried over anhydrous sodium sulfate and concentrated under reduced pressure to afford the crude product, which was purified by column chromatography on silica gel using 5% ethyl acetate in hexane to yield the desired product (0.170 g, 48.7% yield).

Step 4. To a solution of compound 4 (0.280 g, 1.30 mmol) in MeOH (5 mL) were added ammonium carbamate (0.150 g, 1.90 mmol) and [bis(acetoxy)iodo]benzene (0.955 g, 2.90 mmol) at room temperature, and the mixture was stirred for 2 h at the same temperature. The reaction progress was monitored by TLC. After completion, the reaction mixture was concentrated under reduced pressure, and the crude product was purified by column chromatography on silica gel using 1.2% MeOH in DCM to yield intermediate 5 (0.250 g, 78.1% yield).

Step 5. To a solution of compound 5 (0.250 g, 1.00 mmol) in 1,4-dioxane (3 mL) was added hydrogen chloride in dioxane (2.5 mL, 10 V) at 0 °C, and the reaction mixture was stirred for 2 h at room temperature. The reaction progress was monitored by TLC. After completion, the reaction mixture was concentrated under reduced pressure, and the crude product was triturated with pentane to yield intermediate 6 (0.180 g, quantitative yield).

Intermediate 6 was then coupled to Inter-C-(*S*) under standard amide coupling conditions to yield mCMY592.

2. Characterization Data

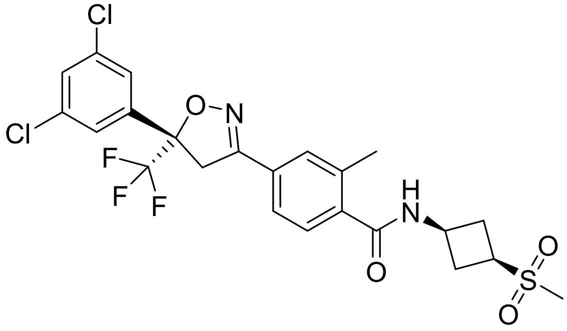

mCMV280

Figure S1. 4-(5-(3,5-dichlorophenyl)-5-(trifluoromethyl)-4,5-dihydroisoxazol-3-yl)-2-methyl-N-((1s,3s)-3-(methylsulfonyl)cyclobutyl)benzamide: 1H NMR (500 MHz, DMSO) δ 8.81 (s, 1H), 7.81 (s, 1H), 7.62 (s, 2H), 7.60 (s, 2H), 7.42 (s, 1H), 4.41 – 4.24 (m, 3H), 3.75 (s, 1H), 2.88 (s, 3H), 2.55 (s, 2H), 2.34 (d, J = 19.3 Hz, 5H). δ LC-MS m/z calcd. C23H22Cl2F3N2O4S+ [M+H]+ = 549.0624, found = 549.1727. DSC MP Onset/Peak= 199/203ºC.

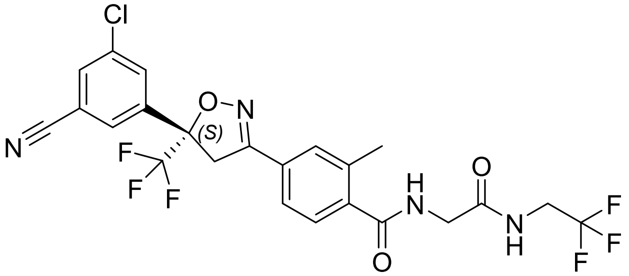

mCMN879

Figure S2. (S)-4-(5-(3-chloro-5-cyanophenyl)-5-(trifluoromethyl)-4,5-dihydroisoxazol-3-yl)-2-methyl-N-(2-oxo-2-((2,2,2-trifluoroethyl)amino)ethyl)benzamide: ^{1}H NMR (400 MHz, MeOD) δ 8.01 – 7.91 (m, 3H), 7.64 (d, *J* = 9.8 Hz, 2H), 7.55 (dd, *J* = 7.9, 1.6 Hz, 1H), 4.31 (dd, *J* = 18.0, 1.6 Hz, 1H), 4.07 (d, *J* = 1.6 Hz, 3H), 4.00 – 3.88 (m, 2H), 2.48 (d, *J* = 1.8 Hz, 3H). LC-MS *m/z* calcd. $C_{23}H_{18}ClF_6N_4O_3^+$ [M+H]$^+$ = 547.0966, found = 547.1295.

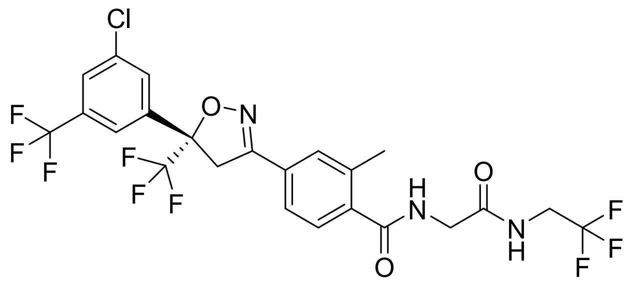

mCMN882

Figure S3. (S)-4-(5-(3-chloro-5-(trifluoromethyl)phenyl)-5-(trifluoromethyl)-4,5-dihydroisoxazol-3-yl)-2-methyl-N-(2-oxo-2-((2,2,2-trifluoroethyl)amino)ethyl)benzamide: ^{1}H NMR (400 MHz, DMSO) δ 8.62 (s, 2H), 8.09 (s, 1H), 7.98 (s, 1H), 7.86 (s, 1H), 7.61 (d, *J* = 6.1 Hz, 2H), 7.48 (s, 1H), 4.41 (s, 2H), 3.94 (d, *J* = 11.4 Hz, 4H), 2.40 (s, 3H). LC-MS *m/z* calcd. $C_{23}H_{18}ClF_9N_3O_3^+$ $[M+H]^+$ = 590.0887, found = 590.0728.

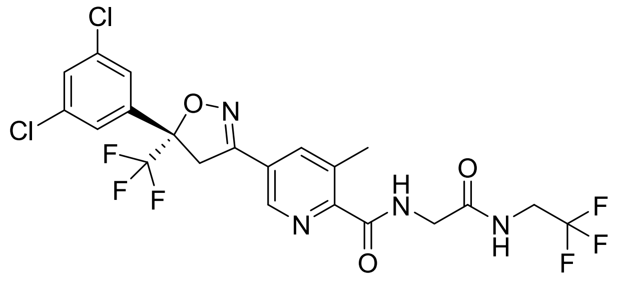

mCMO213

Figure S4. (S)-5-(5-(3,5-dichlorophenyl)-5-(trifluoromethyl)-4,5-dihydroisoxazol-3-yl)-3-methyl-N-(2-oxo-2-((2,2,2-trifluoroethyl)amino)ethyl)picolinamide: ^{1}H NMR (400 MHz, DMSO) δ 8.95 (t, *J* = 6.0 Hz, 1H), 8.77 (d, *J* = 2.3 Hz, 1H), 8.65 (t, *J* = 6.3 Hz, 1H), 8.10 (d, *J* = 2.3 Hz, 1H), 7.84 (t, *J* = 1.9 Hz, 1H), 7.63 (d, *J* = 2.1 Hz, 2H), 4.44 (d, *J* = 30.8 Hz, 2H), 4.00 – 3.88 (m, 4H), 2.60 (s, 3H). LC-MS *m/z* calcd. $C_{21}H_{17}Cl_2F_6N_4O_3^+$ $[M+H]^+$ = 557.0576, found = 557.0367.

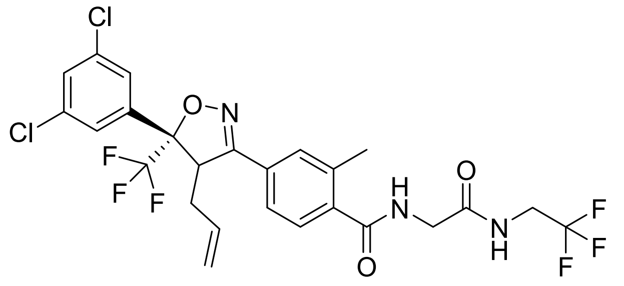

mCMF883

Figure S5. 4-((5S)-4-allyl-5-(3,5-dichlorophenyl)-5-(trifluoromethyl)-4,5-dihydroisoxazol-3-yl)-2-methyl-N-(2-oxo-2-((2,2,2-trifluoroethyl)amino)ethyl)benzamide: ^{1}H NMR (600 MHz, DMSO) δ 8.59 (s, 2H), 7.73 (s, 1H), 7.61 (s, 2H), 7.47 (s, 1H), 7.42 (s, 1H), 7.37 (s, 1H), 5.78 (s, 1H), 5.14 (s, 1H), 5.12 (s, 1H), 3.94 (s, 2H), 3.91 (s, 2H), 3.83 (s, 1H), 3.59 (s, 1H), 3.41 (m, 1H), 2.38 (s, 3H). LC-MS *m/z* calcd. $C_{25}H_{22}Cl_2F_6N_3O_3^+$ $[M+H]^+$ = 596.0937, found = 596.1312.

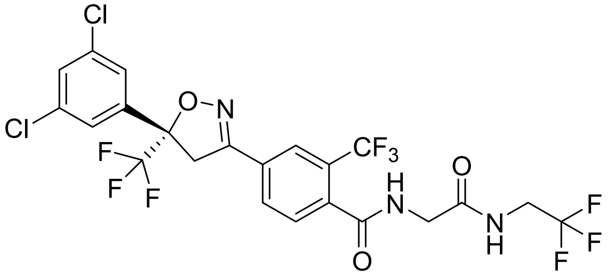

mCMO036

Figure S6. (S)-4-(5-(3,5-dichlorophenyl)-5-(trifluoromethyl)-4,5-dihydroisoxazol-3-yl)-N-(2-oxo-2-((2,2,2-trifluoroethyl)amino)ethyl)-2-(trifluoromethyl)benzamide: ^{1}H NMR (400 MHz, DMSO) δ 8.97 (s, 1H), 8.68 (s, 1H), 8.10 (dd, *J* = 8.0, 1.7 Hz, 1H), 8.03 (d, *J* = 1.8 Hz, 1H), 7.83 (t, *J* = 1.9 Hz, 1H), 7.73 (d, *J* = 8.0 Hz, 1H), 7.64 (d, *J* = 1.9 Hz, 2H), 4.53 (d, *J* = 18.5 Hz, 1H), 4.40 (d, *J* = 18.5 Hz, 1H), 3.94 (s, 4H). LC-MS *m/z* calcd. $C_{22}H_{15}Cl_2F_9N_3O_3^+$ $[M+H]^+$ = 610.0341, found = 610.0007.

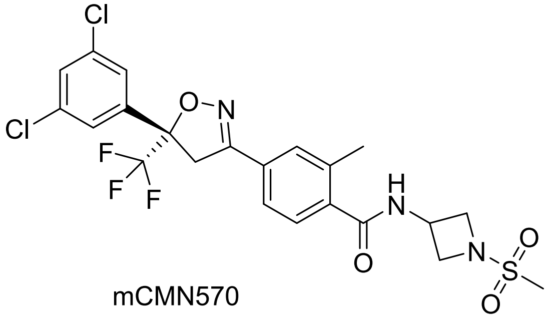

mCMN570

Figure S7. 4-(5-(3,5-dichlorophenyl)-5-(trifluoromethyl)-4,5-dihydroisoxazol-3-yl)-2-methyl-N-(1-(methylsulfonyl)azetidin-3-yl)benzamide: ^{1}H NMR (400 MHz, DMSO) δ 9.03 (d, *J* = 6.8 Hz, 1H), 7.83 – 7.80 (m, 1H), 7.63 (d, *J* = 2.1 Hz, 4H), 7.52 – 7.48 (m, 1H), 4.67 (d, *J* = 6.9 Hz, 1H), 4.37 (t, *J* = 17.9 Hz, 2H), 4.18 – 4.09 (m, 2H), 3.88 (dd, *J* = 8.5, 6.2 Hz, 2H), 3.03 (s, 3H), 2.38 (s, 3H). LC-MS *m/z* calcd. $C_{22}H_{21}Cl_2F_3N_3O_4S^+$ $[M+H]^+$ = 550.0576, found = 550.0635.

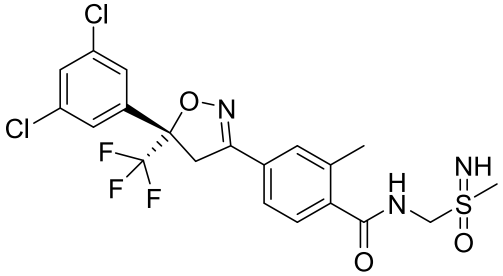

mCMV503

Figure S8. 4-(5-(3,5-dichlorophenyl)-5-(trifluoromethyl)-4,5-dihydroisoxazol-3-yl)-2-methyl-N-((S-methylsulfonimidoyl)methyl)benzamide: ^{1}H NMR (400 MHz, DMSO) δ 9.11 (s, 1H), 7.82 (s, 1H), 7.63 (s, 4H), 7.47 (s, 1H), 4.64 (s, 1H), 4.62 (s, 1H), 4.36 (s, 1H), 4.32 (s, 1H), 3.27 (s, 3H), 2.39 (s, 3H). LC-MS *m/z* calcd. $C_{20}H_{19}Cl_2F_3N_3O_3S^+$ $[M+H]^+$ = 508.0471, found = 508.0327.

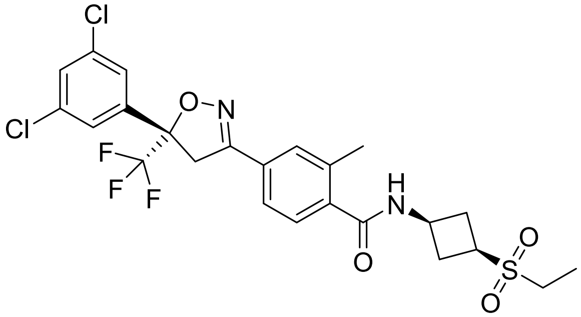

mCMY265

Figure S9. 4-(5-(3,5-dichlorophenyl)-5-(trifluoromethyl)-4,5-dihydroisoxazol-3-yl)-N-((1s,3s)-3-(ethylsulfonyl)cyclobutyl)-2-methylbenzamide: ^{1}H NMR (400 MHz, DMSO) δ 8.84 (d, *J* = 7.2 Hz, 1H), 7.82 (t, *J* = 1.9 Hz, 1H), 7.65 – 7.52 (m, 4H), 7.43 (d, *J* = 7.9 Hz, 1H), 4.38 – 4.33 (m, 3H), 3.78 (m, 1H), 2.99 (q, *J* = 7.5 Hz, 2H), 2.56 (m, 2H), 2.35 – 2.33 (m, 5H), 1.21 – 1.16 (t, *J* = 7.5 Hz, 3H). LC-MS *m/z* calcd. $C_{24}H_{24}Cl_2F_3N_2O_4S^+$ $[M+H]^+$ = 563.0780, found = 563.1334.

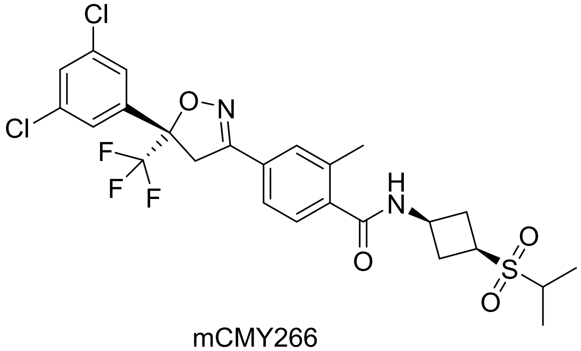

mCMY266

Figure S10. 4-(5-(3,5-dichlorophenyl)-5-(trifluoromethyl)-4,5-dihydroisoxazol-3-yl)-N-((1s,3s)-3-(isopropylsulfonyl)cyclobutyl)-2-methylbenzamide: ^{1}H NMR (400 MHz, DMSO) δ 8.84 (d, *J* = 7.2 Hz, 1H), 7.82 (t, *J* = 1.9 Hz, 1H), 7.67 – 7.54 (m, 4H), 7.43 (d, *J* = 7.9 Hz, 1H), 4.43 – 4.22 (m, 3H), 3.85 (p, *J* = 8.8 Hz, 1H), 3.14 (p, *J* = 6.9 Hz, 1H), 2.56 (m, 2H), 2.43 – 2.30 (m, 5H), 1.21 (d, *J* = 6.9 Hz, 6H). LC-MS *m/z* calcd. $C_{25}H_{26}Cl_2F_3N_2O_4S^+$ $[M+H]^+$ = 577.0937, found = 577.0795.

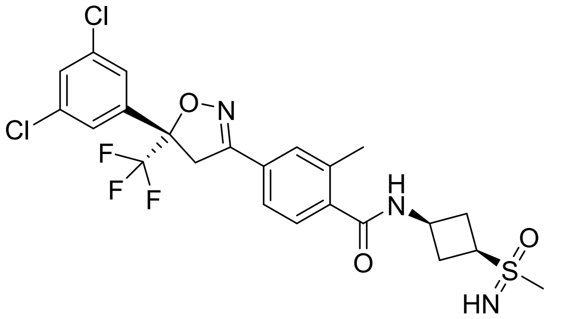

mCMY592

Figure S11. 4-(5-(3,5-dichlorophenyl)-5-(trifluoromethyl)-4,5-dihydroisoxazol-3-yl)-2-methyl-N-((1S,3s)-3-((R)-S-methylsulfonimidoyl)cyclobutyl)benzamide: ^{1}H NMR (400 MHz, DMSO) δ 8.75 (d, *J* = 7.3 Hz, 1H), 7.82 (t, *J* = 1.8 Hz, 1H), 7.68 – 7.54 (m, 4H), 7.42 (d, *J* = 7.8 Hz, 1H), 4.44 – 4.24 (m, 3H),

3.67 (p, *J* = 8.8 Hz, 1H), 3.58 (s, 1H), 2.78 (s, 3H), 2.57 – 2.53 (m, 1H), 2.36 (m, 5H). LC-MS *m/z* calcd. $C_{23}H_{23}Cl_2F_3N_3O_3S^+$ $[M+H]^+$ = 548.0784, found = 548.0821.

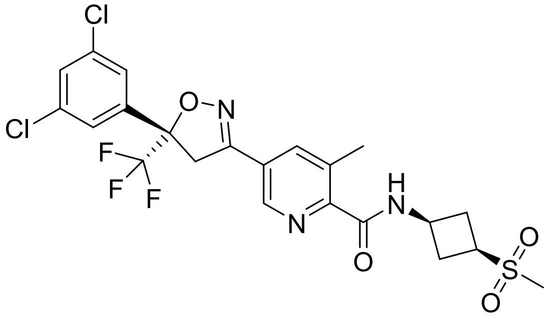

mCMX946

Figure S12. 5-(5-(3,5-dichlorophenyl)-5-(trifluoromethyl)-4,5-dihydroisoxazol-3-yl)-3-methyl-N-((1s,3s)-3-(methylsulfonyl)cyclobutyl)picolinamide: ^{1}H NMR (400 MHz, DMSO) δ 9.10 (d, *J* = 7.7 Hz, 1H), 8.75 (s, 1H), 8.06 (s, 1H), 7.83 (s, 1H), 7.63 (s, 2H), 4.57 – 4.33 (m, 3H), 3.83 – 3.69 (m, 1H), 2.90 (s, 3H), 2.58 – 2.52 (m, 5H),j 2.42 (m, 2H). LC-MS *m/z* calcd. $C_{22}H_{21}Cl_2F_3N_3O_4S^+$ $[M+H]^+$ = 550.0576, found = 550.1016.

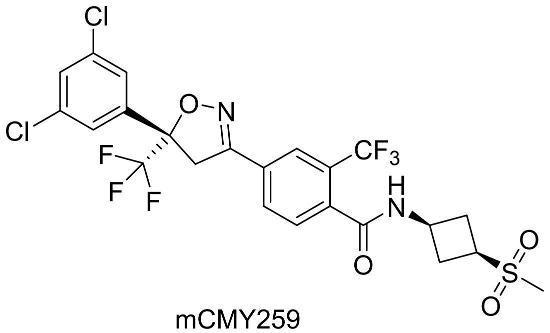

mCMY259

Figure S13. 4-(5-(3,5-dichlorophenyl)-5-(trifluoromethyl)-4,5-dihydroisoxazol-3-yl)-N-((1s,3s)-3-(methylsulfonyl)cyclobutyl)-2-(trifluoromethyl)benzamide: ^{1}H NMR (400 MHz, DMSO) δ 9.09 (d, *J* = 7.2 Hz, 1H), 8.10 – 7.97 (m, 2H), 7.83 (t, *J* = 1.9 Hz, 1H), 7.70 – 7.59 (m, 3H), 4.67 – 4.29 (m, 3H), 3.77 (p, *J* = 8.7 Hz, 1H), 2.88 (s, 3H), 2.57 (m, 2H), 2.30 (m, 2H). LC-MS *m/z* calcd. $C_{23}H_{19}Cl_2F_6N_2O_4S^+$ $[M+H]^+$ = 603.0341, found = 603.0659.

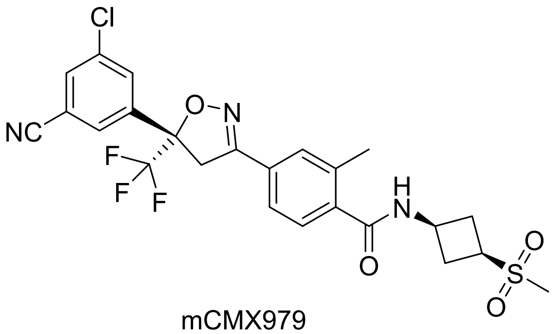

mCMX979

Figure S14. 4-(5-(3-chloro-5-cyanophenyl)-5-(trifluoromethyl)-4,5-dihydroisoxazol-3-yl)-2-methyl-N-((1s,3s)-3-(methylsulfonyl)cyclobutyl)benzamide: ^{1}H NMR (400 MHz, DMSO) δ 8.83 (d, *J* = 7.2 Hz, 1H),

8.25 (t, *J* = 1.7 Hz, 1H), 8.06 (s, 1H), 7.98 (d, *J* = 2.3 Hz, 1H), 7.58 (m, 2H), 7.44 (d, *J* = 7.9 Hz, 1H), 4.44 – 4.29 (m, 3H), 3.76 (p, *J* = 8.9 Hz, 1H), 2.88 (s, 3H), 2.63 – 2.52 (m, 2H), 2.39 – 2.28 (m, 5H). LC-MS *m/z* calcd. $C_{24}H_{22}ClF_3N_3O_4S^+$ $[M+H]^+$ = 540.0966, found = 540.1562.

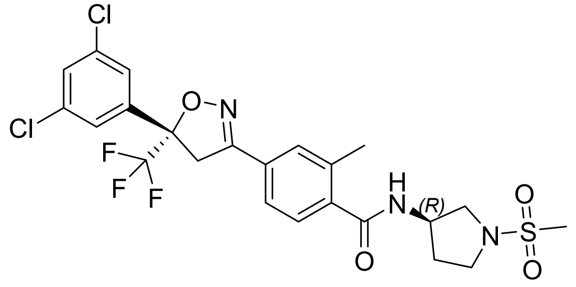

mCMV092

Figure S15. 4-((S)-5-(3,5-dichlorophenyl)-5-(trifluoromethyl)-4,5-dihydroisoxazol-3-yl)-2-methyl-N-((R)-1-(methylsulfonyl)pyrrolidin-3-yl)benzamide: ^{1}H NMR (400 MHz, DMSO) δ 8.64 (d, *J* = 6.5 Hz, 1H), 7.82 (t, *J* = 1.9 Hz, 1H), 7.65 – 7.57 (m, 4H), 7.44 (d, *J* = 7.8 Hz, 1H), 4.45 (m, 1H), 4.34 (q, *J* = 18.4 Hz, 2H), 3.56 (dd, *J* = 10.3, 6.7 Hz, 1H), 3.43 – 3.35 (m, 2H), 3.17 (dd, *J* = 10.3, 5.0 Hz, 1H), 2.92 (s, 3H), 2.36 (s, 3H), 2.18 (dq, *J* = 13.8, 6.8 Hz, 1H), 1.92 (dq, *J* = 12.9, 6.4 Hz, 1H). LC-MS *m/z* calcd. $C_{23}H_{23}Cl_2F_3N_3O_4S^+$ $[M+H]^+$ = 564.0733, found = 564.0861.

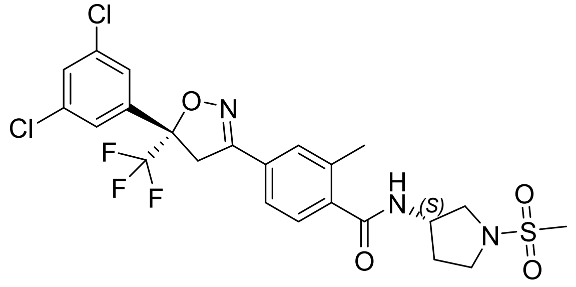

mCMV074

Figure S16. 4-((S)-5-(3,5-dichlorophenyl)-5-(trifluoromethyl)-4,5-dihydroisoxazol-3-yl)-2-methyl-N-((S)-1-(methylsulfonyl)pyrrolidin-3-yl)benzamide: ^{1}H NMR (400 MHz, DMSO) δ 8.64 (d, *J* = 6.6 Hz, 1H), 7.82 (t, *J* = 1.8 Hz, 1H), 7.66 – 7.57 (m, 4H), 7.44 (d, *J* = 7.8 Hz, 1H), 4.34 (m, 1H), 4.41 – 4.24 (q, *J* = 18.4 Hz, 2H), 3.56 (dd, *J* = 10.3, 6.6 Hz, 1H), 3.43 – 3.35 (m, 2H), 3.16 (dd, *J* = 10.3, 4.9 Hz, 1H), 2.92 (s, 3H), 2.36 (s, 3H), 2.18 (dq, *J* = 13.6, 6.8 Hz, 1H), 1.92 (dq, *J* = 13.0, 6.4 Hz, 1H). LC-MS *m/z* calcd. $C_{23}H_{23}Cl_2F_3N_3O_4S^+$ $[M+H]^+$ = 564.0733, found = 564.0479.

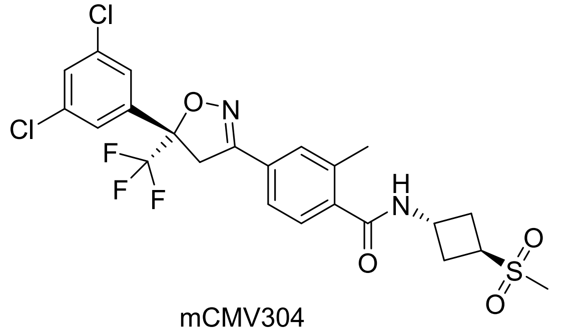

mCMV304

Figure S17. 4-(5-(3,5-dichlorophenyl)-5-(trifluoromethyl)-4,5-dihydroisoxazol-3-yl)-2-methyl-N-((1r,3r)-3-(methylsulfonyl)cyclobutyl)benzamide: ^{1}H NMR (400 MHz, DMSO) δ 8.77 (d, *J* = 7.3 Hz, 1H), 7.82 (t, *J* = 1.9 Hz, 1H), 7.67 – 7.57 (m, 4H), 7.46 (d, *J* = 7.9 Hz, 1H), 4.52 (p, *J* = 7.9 Hz, 1H), 4.34 (q, *J* = 18.4 Hz, 2H), 3.85 (m, 1H), 2.96 (s, 3H), 2.74 – 2.64 (m, 2H), 2.45 (m, 2H), 2.36 (s, 3H). δ LC-MS *m/z* calcd. $C_{23}H_{22}Cl_2F_3N_2O_4S^+$ $[M+H]^+$ = 549.0624, found = 549.0728.

3. DMPK Assays

Plasma Protein and Brain Homogenate Binding Studies

Studies were carried out at WuXi AppTec (Shanghai, China). For LC/MS/MS analysis, test and control compounds were quantified using peak area ratio of analyte and internal standard. Protein binding in plasma was determined using equilibrium dialysis. Compounds were tested at 2 µM in triplicate, and concentrations were quantified by LC/MS/MS.

Hepatocyte stability studies

Studies were carried out at Aragen Life Sciences (India). Stability of test article (1 µM) was monitored at 0, 15, 30, 60, 90, and 120 min in the presence of hepatocytes (source = Thermo Fisher Scientific, cell density = 0.4 million/mL), and the data (%Remaining, half-life, Clearance) generated in duplicate (analysis = LCMS/MS quantification of test article using an internal standard).

MDCK-MDR1

Studies were carried out at Bioduro (Shanghai, China). Compounds were tested at 10 µM in duplicate, with atenolol, propranolol and digoxin used as controls, and concentrations were quantified by LC/MS/MS.

hERG manual patch clamp assay

Compounds were tested for effect on hERG potassium channels using the whole-cell patch clamp technique with a Multiclamp 700 patch-clamp amplifier (Molecular Devices, USA) at S20 WuXi AppTec (Shanghai, China). CHO cells stably expressing hERG potassium channels from Aviva Biosciences (San Diego, CA) were tested with compounds at five concentrations, in a three-fold serial dilution starting at 30 µM, compared to vehicle (negative) control and amitriptyline (WuXi AppTec, Shanghai, China) (positive) controls. Percentage of control (vehicle) values were calculated in duplicate for each concentration of drug, and data analysis was performed using Clampfit (V10.7, Molecular Deveices) and GraphPad Prism.

Mini-Ames

Mini-Ames study was done at WuXi AppTec (Shanghai, China) to evaluate the test article mCMV280, for its ability to induce reverse mutations both in the presence and absence of S9 mix at the histidine locus in the genome of four strains of Salmonella typhimurium (TA98, TA100, TA1535, and TA97a) and at the tryptophan locus in the genome of Escherichia coli WP2 uvrA (pKM101). The assay was conducted in the presence and absence of S9 mix along with the concurrent negative/solvent control (DMSO) using six wells and positive controls using three wells. The tested dose levels in the mutagenicity assay with five tester strains in the presence and absence of S9 mix were 1.5, 4, 10, 25, 64, 160, 400, and 1000 µg per well, three wells per dose. The study was conducted using fresh cultures of the bacterial strains and fresh test article formulations.

In vitro micronucleus induction

In vitro microwell micronucleus screening assay in Chinese hamster ovary cells (CHO-WBL, Merck Research Laboratories, USA) was carried out at WuXi AppTec (Shanghai, China). Clastogenicity/aneugenicity was measured by the extent of micronucleus formation with and S21 without exogenous metabolic activation (Aroclor 1254 induced rat liver S9, Molecular Toxicology (Boone, NC). Cultures of CHO-WBL cells in Microwell 8-well chamber slides (Thermo Fisher Scientific Inc.) were exposed in duplicate to multiple concentrations of test article as well as to positive (cyclophosphamide monohydrate, mitomycin C) and solvent controls. In the S9 activated test system (test article concentrations: 50, 100, 245, 491.5 µg/mL), exposure was for 3 h; in the non-activated test system (test article concentrations: 50, 100, 240, 260, 280, 300, 320, 340, 360, 380, 400, 420, 440, 460, 480 µg/mL), treatment was for 3 h and for 24 h. Cells were fixed and stained with acridine orange, and 2000 binucleated cells (1000 binucleated cells/culture) were scored for each test and control article concentration.

In vitro safety profiling assays

Electrophysiological assays (cardiac panel) were conducted to profile mCMV280 for activities on the ion channel targets (voltage-Gated Sodium: HEK-Nav1.5, voltage-Gated Calcium: HEK-Cav1.2, voltage-Gated Potassium: CHO-hERG) using the QPatch electrophysiological platform at Eurofins Panlabs Inc.

B/P and $K_{p,uu}$ ratio

The B/P ratio in mice was calculated by taking the ratio of the brain concentration over the plasma concentration at $T_{max,brain}$ or the ratio of the brain AUC_{all} over the plasma AUC_{all}. The B/P ratio was calculated after take down in beagles and cynos to give the terminal B/P ratio. The $K_{p,uu}$ is calculating in the same way as B/P but takes into account the plasma protein and brain homogenate binding. The ratios are calculated using the unbound fraction ($f_{u,brain}/f_{u,plasma}$).

In vivo assays

Animal Study: Animal Care and Compound Application.

Animal experimental procedures were approved by the Institutional Animal Care and Use Committee (IACUC) of the respective study locations. Pharmacokinetic studies were conducted at Aragen (India), Calibr (San Diego, CA), GVK (India), WuXi (China), or Pharmaron Inc. (China). IACUC codes SZ20200529, PK-CM-0220202, and CR24-001.

Pharmacokinetics in animals [CD1-mouse, SD-rat, beagle dog, or cynomolgus monkeys]

For all IV pharmacokinetic studies, three fasted animals per study group were administered the test article as a solution in 75% PEG 300/25%D5W. Blood samples were collected at 0.083, 0.5, 1, 3, 5, 8, 24, 47, 72, and up to 336 h for IV studies if needed to better capture half-life. For mouse PO pharmacokinetic studies with brain concentration measurements, 3 fasted animals per study group were administered the test article as a solution in 75% PEG 300/25%D5W. 1 group of 3 were terminated for each brain concentration time point, typically meaning 12 animals total were dosed. Blood samples were collected at 0.5, 1, 3, 5, 8, 24, 48 (optional) and 72 (optional) post-dosing for mouse PO studies. Non-perfused brain concentration measurements were taken at 1, 3, 8, and 24 hrs post dose. For PO pharmacokinetic studies monitoring just plasma concentrations dosed in mice and rats, three fasted animals per study group were administered the test article as a suspension in 0.5% MC + 0.5% Tween 80 and blood samples were collected at 0.5, 1, 3, 5, 8, 24, 48, 72, 96, and 120 hrs. For PO pharmacokinetic studies dosed in dogs, three fasted animals per study group were administered the test article as a suspension in 0.5% MC +

0.5% Tween 80 and blood samples were collected at 0.5, 1, 3, 5, 8, 24, 72, 120, 168, 240, 288, 336, 384, 432, 504, 576, and 648 hrs. For PO pharmacokinetic studies dosed in Cyno monkeys, three fasted animals per study group were administered the test article as a suspension in 0.1% MC + 0.2% Tween 80 and blood samples were collected at 0.5, 1, 3, 5, 8, 24, 48, 72, 96, 120, 144, 168, 216, 240, 264, 288, 312, and 336 hrs. The blood samples were centrifuged to obtain the plasma, which was stored below -20°C until analysis. Plasma and brain concentrations were determined by liquid chromatography/tandem mass spectrometry (LC−MS/MS). The PK parameters were determined by non-compartmental methods using WinNonLin (v6.1 or higher version, Certara Inc.).

4. Additional Figures/Tables

	mCMV280	Fluralaner
**Pharmacokinetic Parameters Following Single PO dose** (mouse/rat/dog/cyno)		
CL (mL/min/kg])	0.59 / 0.86 / 0.18/ 0.15	2.18 /ND / 0.40 / 0.33
DN AUC_{inf}	18080 / 15649 / 2334 / ND	4519 / ND / 12079 / 10375
$T_{1/2}$ (h)	46.0 / 31.86 / 250 / 103	12.6 / ND / 215 / 90.5
Mouse Brain Exposure C_{max} (nM)	1830	8782
Mouse B/P, $K_{p,uu}$ at $C_{max,brain}$	0.12, 0.0015	0.95, 0.017

Table S1. Comparative *in vivo* performance of lead mCMV280 vs. fluralaner

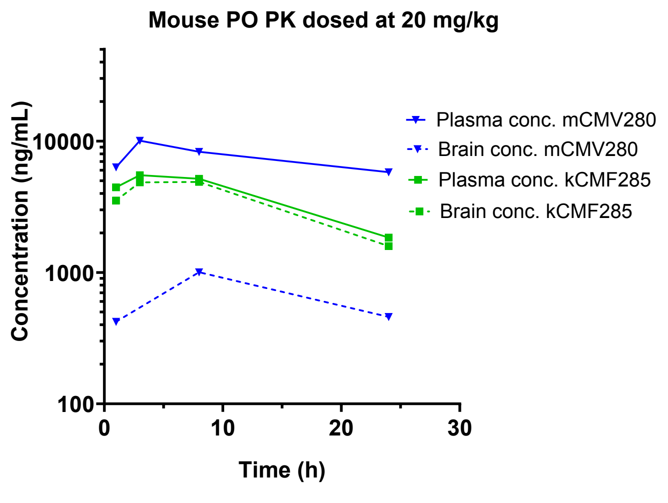

Figure S18. Mouse oral PK of mCMV280 vs kCMF285 measuring brain and plasma concentration Both compounds were dosed at 20 mg/kg as solutions in 75%PEG300/25%D5W.

5. Characterization and spectral data of mCMV280

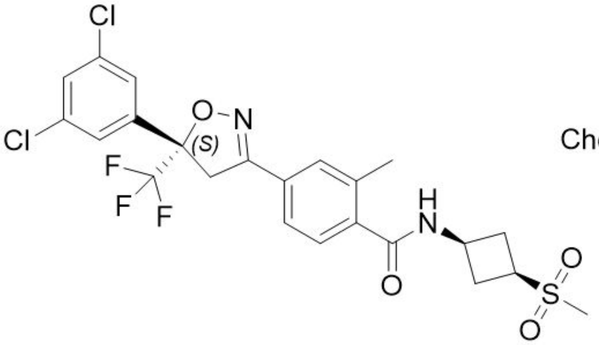

mCMV280

Chemical Formula: $C_{23}H_{21}Cl_2F_3N_2O_4S$
Exact Mass: 548.0551
Molecular Weight: 549.3862

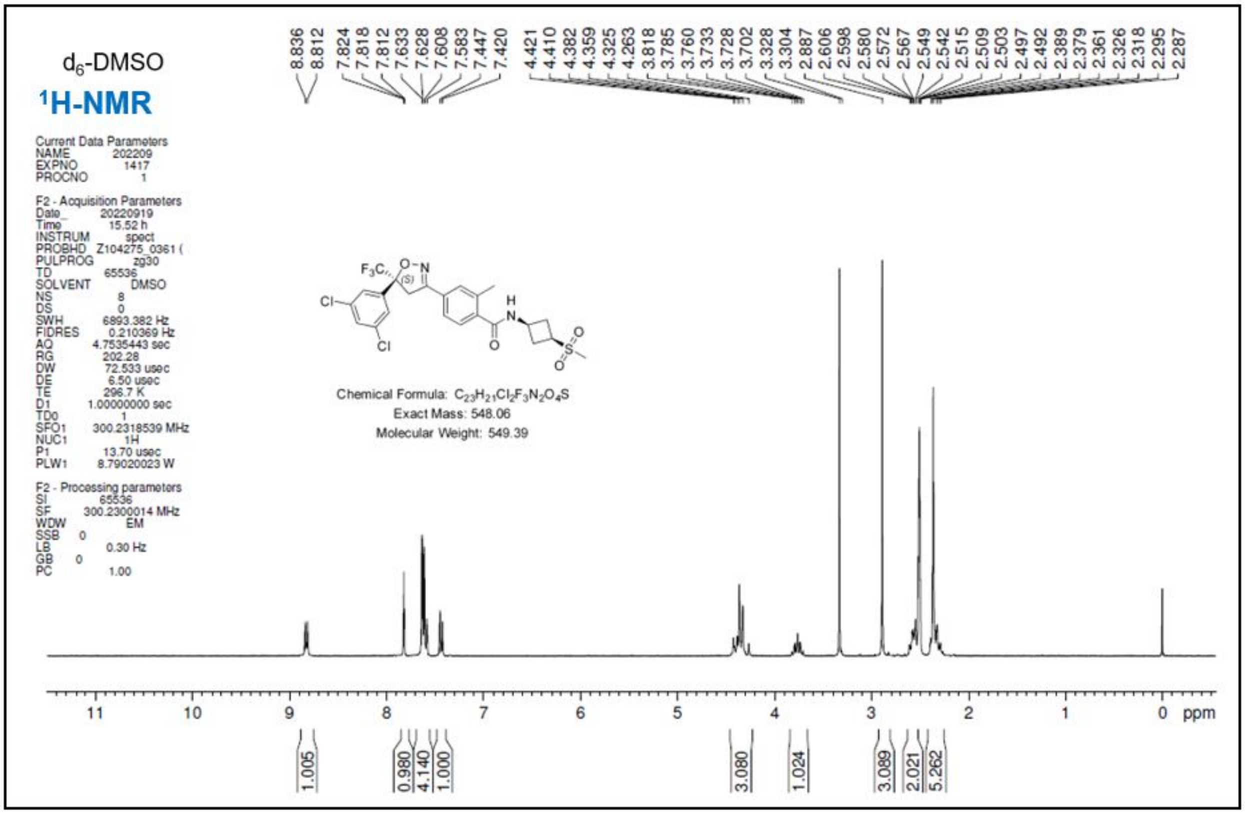

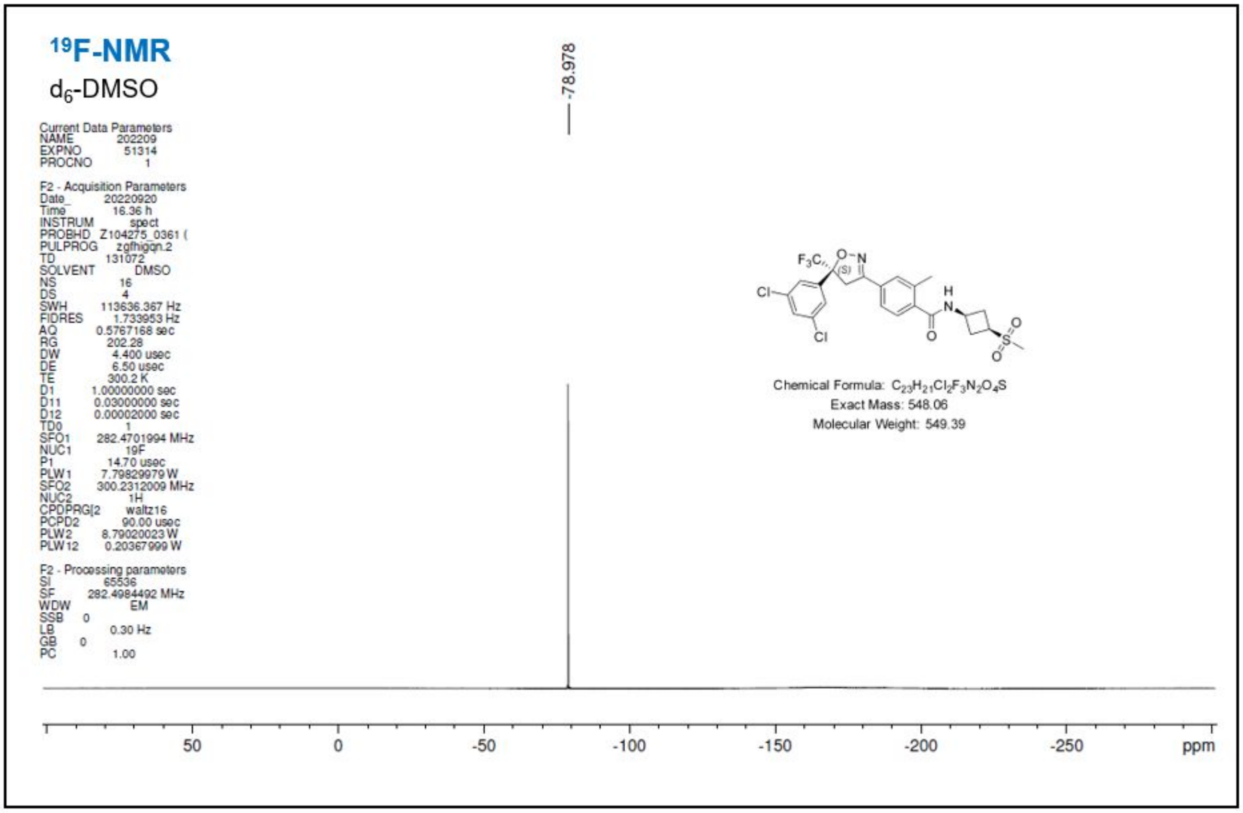

Figure S19. Characterization and spectral data of mCMV280

Chiral SFC-MS Analysis Report mCMV280

Acquired by : System Administrator
Data File : SFC_MS01-PH-CLR-SPC-2022-31-0-1(EB2211004-009A1)1T.lcd
Method Filename : Scouting_Method-3.0.lcm
Date Acquired : 9/19/2022 12:22:56 PM
Vial : 1-77
Injection Volume : 1 uL
Column Name : Lux-4 100x4.6mm 3.0um
Co Solvent : MeOH (20mM NH3)

Oven Temperature : 35 C
Total Flow : 3.0000 mL/min
Start Conc. of Pump B : 10.0%
BPR Pressure : 15.00 MPa
BPR Temperature : 50 C
LC Gradient Program :

Time	Module	Command	Value
2.50	Pumps	B.Conc	50
2.51	Pumps	B.Conc	50
3.70	Pumps	B.Conc	50
3.71	Pumps	B.Conc	10
4.00	Controller	Stop	

Chromatogram

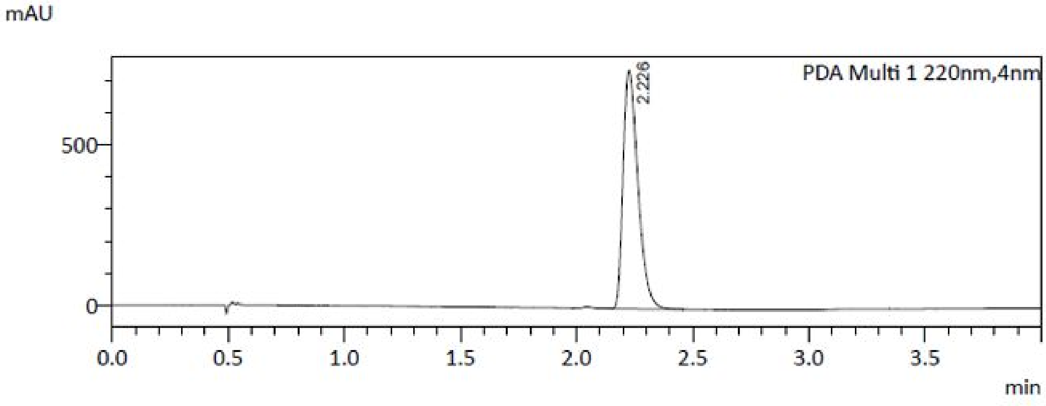

PDA Ch1 220nm

Peak#	Ret. Time	Height	Area	Area%	Resolution(JP)
1	2.226	744259	3321758	100.000	--
Total			3321758	100.000	

Figure S20. Chirality report for mCMV280

LCMS Conditions and Mass Spectrum mCMV280

<<Sample Information>>
Acquired by : System Administrator
Sample Name : LCMS10-PH-CLR-SPC-2022-31-0-1(EB2211004-009A1)1T
Sample ID : LCMS102152795
Injection Volume : 1 uL
Data File : LCMS10-PH-CLR-SPC-2022-31-0-1(EB2211004-009A1)1T.lcd
Method File : 5%-95%B-3min-1.5.lcm
Date Acquired : 9/19/2022 2:53:11 PM

<<Column>>
HALO RP-Amide, 50*3.0 mm, 2.7 um

<<Mobile Phase>>
Mobile Phase A : Water/0.05%TFA
Mobile Phase B : Acetonitrile/0.05%TFA

<<LC Time Program>>

Time	Module	Command	Value
2.00	Pumps	B.Conc	95
2.80	Pumps	B.Conc	95
2.81	Pumps	B.Conc	5
3.00	Controller	Stop	

<<Instrument>>
Shimadzu LCMS2020

<<Pump>>
Mode : Binary gradient
Pump A : LC-20ADXR
Pump B : LC-20ADXR
Total Flow : 1.5000 mL/min
B Conc. : 5.0 %

<<Oven>>
Oven Temperature : 40 C

<<PDA>>
PDA Model : SPD-M20A
Lamp : D2
Start Wavelength : 190 nm
End Wavelength : 800 nm

<<Interface>>
Interface :ESI
DL Temperature :250 C
Nebulizing Gas Flow :1.50 L/min
Heat Block :250 C
Drying Gas :On
15.00 L/min

<<MS Parameter>>
Initial Valve Position :-
--Segment 1 Event 1--
Start Time :0.00 min
End Time :3.00 min
Acquisition Mode :Scan
Polarity :Positive
Event Time :0.40 sec
Detector Voltage :+1.15 kV
Threshold :1000
Start m/z :90.00
End m/z :900.00
Scan Speed :2143 u/sec
Interface Voltage :4.50 kV
DL Volt. :Use the Data in the Tuning File
Qarray DC Voltage :Use the Data in the Tuning File
Qarray DC Voltage :Use the Data in the Tuning File

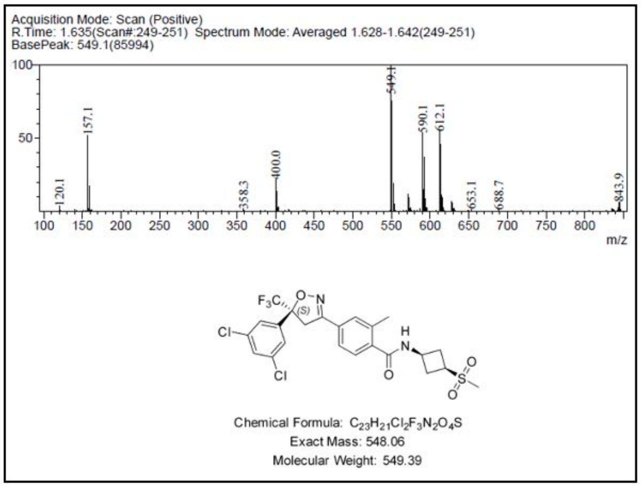

Figure S21. LCMS and mass spectra of mCMV280

Quantitative ^{1}H-NMR in d_6-DMSO of mCMV280

IS 1H	Sam 1H	MW of SAM	MW of IS	Wt. IS	Wt. Sam	Intensity Sam	Intensity IS	Purity IS	Weight %
3	1	549.39	168.19	10.19	25.08	1	4.062	99.00	97.04

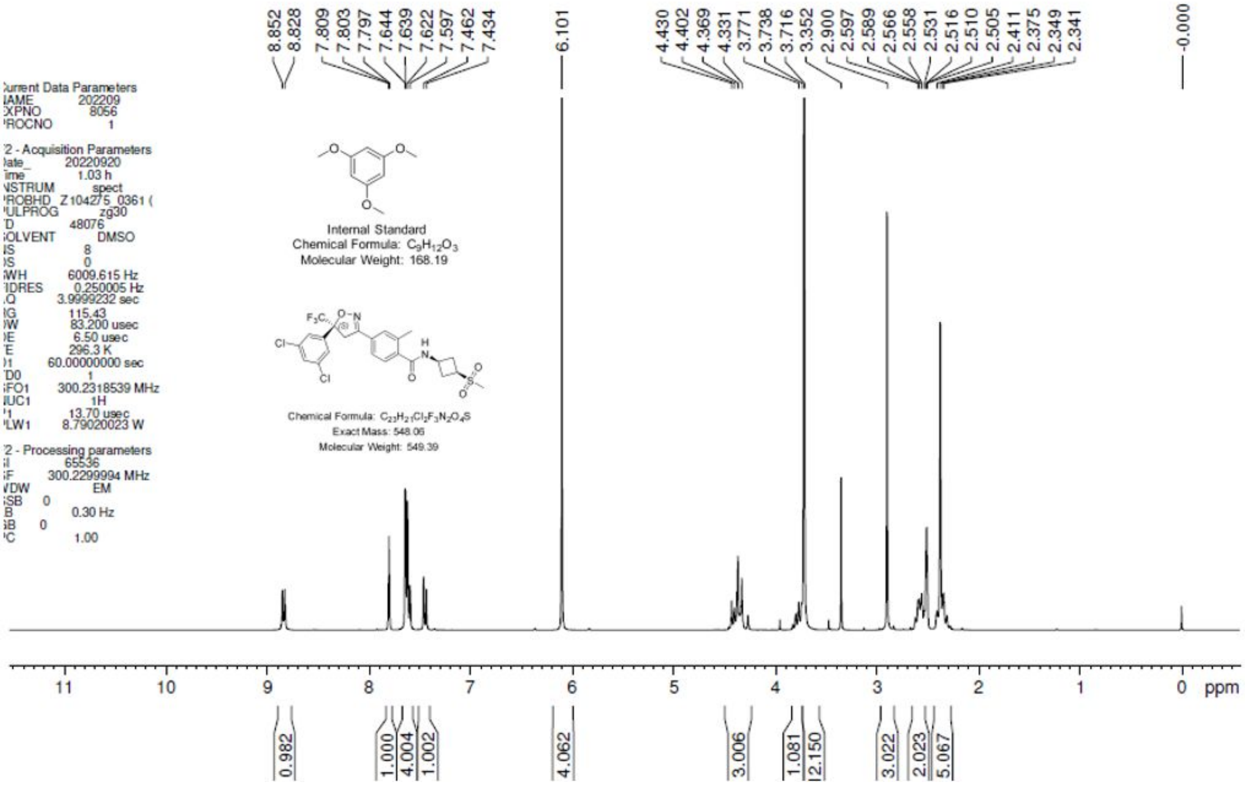

DSC of mCMV280

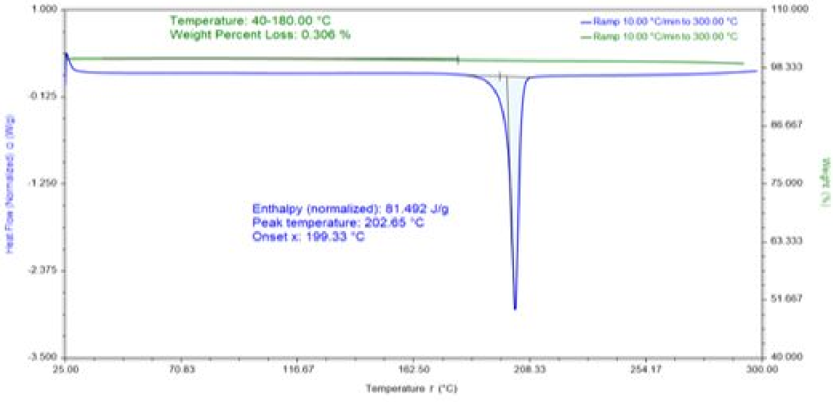

Figure S23. H-NMR of mCMV280

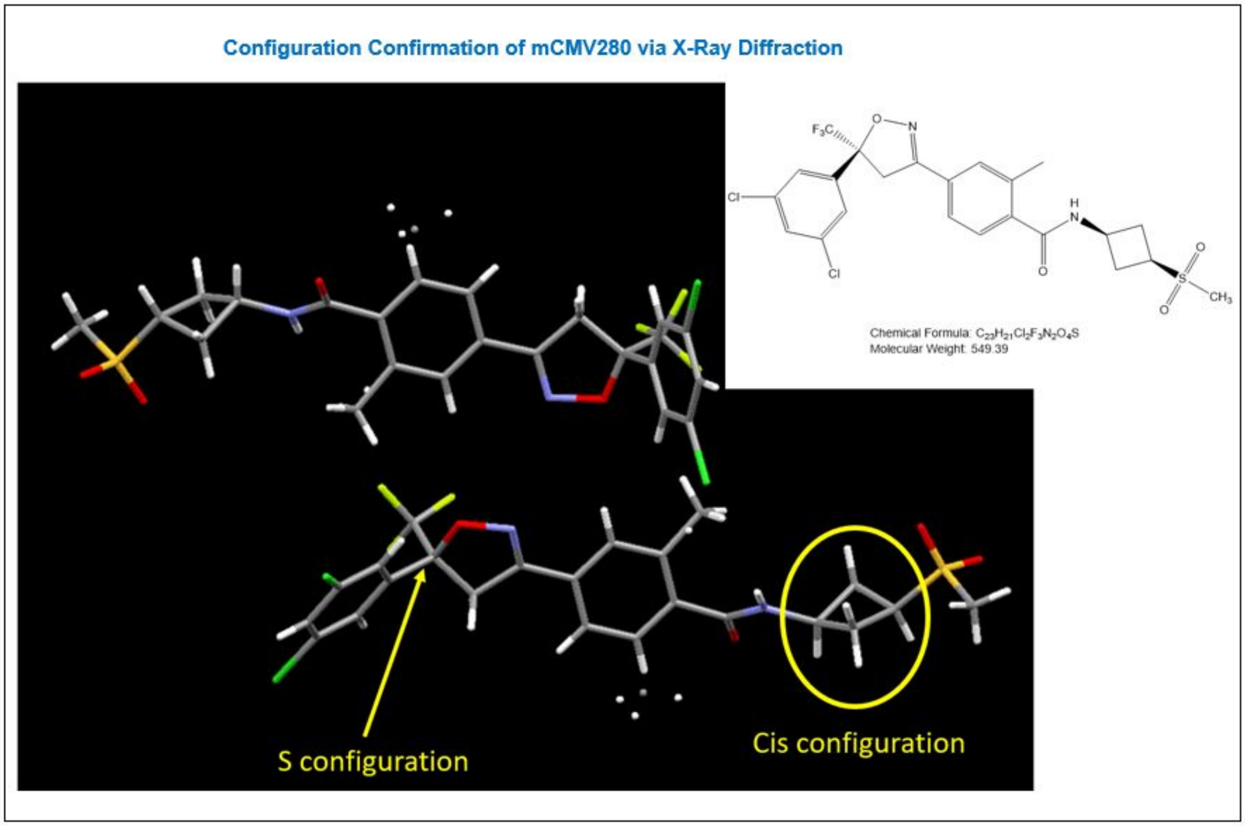

Figure S24. Single Crystal Determination of mCMV280. Isomeric conformation of of mCMV280

Table S2. Crystal data and structure refinement for mCMV280.

Empirical formula	$C_{23}H_{21}N_2O_4F_3SCl_2$
Formula weight	549.38
Temperature/K	180.00(10)
Crystal system	monoclinic
Space group	$P2_1$
a/Å	12.4285(7)
b/Å	9.2517(4)
c/Å	23.8383(8)
α/°	90
β/°	92.302(4)
γ/°	90
Volume/Å^3	2738.8(2)
Z	4
ρ_{calc}g/cm^3	1.332
μ/mm^{-1}	3.299
F(000)	1128.0
Crystal size/mm^3	0.48 × 0.03 × 0.02
Radiation	Cu Kα (λ = 1.54184)
2θ range for data collection/°	3.71 to 144.42
Index ranges	$-15 \leq h \leq 15$, $-11 \leq k \leq 11$, $-28 \leq l \leq 29$
Reflections collected	25725
Independent reflections	9782 [R_{int} = 0.0846, R_{sigma} = 0.0742]
Data/restraints/parameters	9782/48/597
Goodness-of-fit on F^2	1.049
Final R indexes [I>=2σ (I)]	R_1 = 0.0923, wR_2 = 0.2282
Final R indexes [all data]	R_1 = 0.1171, wR_2 = 0.2441
Largest diff. peak/hole / e Å^{-3}	0.46/-0.35
Flack parameter	0.07(3)

Table S3. Fractional Atomic Coordinates (×10^4) and Equivalent Isotropic Displacement Parameters (Å^2×10^3) for CMV280.

Atom	*x*	*y*	*z*	U(eq)
Cl1	5544(10)	10216(6)	4400(4)	308(5)
Cl2	7755(5)	5584(9)	5059(2)	226(3)
S1	3607(2)	3493(3)	-2107.8(9)	82.9(7)
F1	3420(10)	3414(16)	3399(4)	219(6)
F2	3435(10)	4751(15)	4123(4)	202(5)
F3	4677(10)	3129(11)	4034(4)	187(5)
O1	2801(6)	4242(10)	-1851(3)	103(2)
O2	3285(7)	2253(10)	-2440(3)	117(3)
O3	5579(6)	1370(6)	221(3)	94(2)
O4	4007(8)	6128(11)	3167(3)	131(3)
N1	4898(6)	3400(8)	-165(2)	79(2)
N2	3968(8)	5766(12)	2595(4)	120(3)
C1	4311(9)	4693(12)	-2530(4)	106(4)
C2	4561(6)	2941(8)	-1586(3)	81(3)
C3	4150(7)	2163(9)	-1084(3)	78(2)
C4	5053(7)	2895(8)	-740(2)	82(3)
C5	5102(9)	4038(8)	-1201(3)	87(3)
C6	5185(7)	2564(7)	281(3)	72(2)
C7	5017(5)	3192(6)	845.2(16)	74(2)
C8	5866(4)	3048(6)	1239(2)	76(2)
C9	5765(4)	3570(7)	1781(2)	79(2)
C10	4816(5)	4237(7)	1929.3(16)	85(3)
C11	3967(4)	4381(7)	1536(2)	81(2)
C12	4068(4)	3858(7)	994(2)	79(3)
C13A	3071(7)	3953(16)	605(5)	89(4)

Table S3. Fractional Atomic Coordinates (×10[4]) and Equivalent Isotropic Displacement Parameters (Å[2]×10[3]) for CMV280.

Atom	*x*	*y*	*z*	U(eq)
C13B	6984(19)	2470(50)	1130(20)	89(4)
C14	4691(8)	4821(10)	2506(2)	94(3)
C15	5418(8)	4519(12)	3007(3)	95(3)
C16	4751(7)	5177(9)	3457(3)	119(4)
C17	4074(9)	4115(11)	3762(4)	160(7)
C18	5370(7)	6048(9)	3892(3)	117(4)
C19	6190(8)	5450(6)	4232(4)	123(4)
C20	6757(7)	6309(12)	4620(4)	156(6)
C21	6504(9)	7766(11)	4669(4)	193(9)
C22	5685(11)	8364(6)	4330(5)	184(9)
C23	5117(8)	7506(10)	3941(4)	175(8)
Cl3	2521(3)	9365(6)	333.9(14)	139.6(14)
Cl4	-1594(3)	10846(5)	560.2(15)	142.1(14)
S2	1551(2)	7319(4)	7062.6(10)	99.9(9)
F4	104(8)	4542(10)	754(3)	149(3)
F5	-1460(8)	5258(11)	953(3)	155(3)
F6	-643(11)	3734(10)	1484(3)	188(5)
O5	1948(8)	8571(13)	7357(3)	139(4)
O6	2291(7)	6508(14)	6760(3)	145(4)
O7	-637(7)	9528(6)	4803(2)	96(2)
O8	1004(7)	5587(10)	1719(3)	119(3)
N3	89(8)	7494(8)	5178(2)	92(3)
N4	1112(7)	5803(10)	2317(3)	101(3)
C24	869(9)	6216(13)	7533(4)	114(4)
C25	506(6)	7911(8)	6599(3)	80(3)

Table S3. Fractional Atomic Coordinates (×10^4) and Equivalent Isotropic Displacement Parameters (Å^2×10^3) for CMV280.

Atom	*x*	*y*	*z*	U(eq)
C26	-58(9)	6832(9)	6205(3)	88(3)
C27	-53(8)	7972(8)	5755(2)	90(3)
C28	873(9)	8701(12)	6078(3)	102(4)
C29	-232(8)	8345(8)	4745(2)	81(3)
C30	-80(5)	7739(6)	4168.6(15)	75(3)
C31	-942(4)	7863(7)	3781(2)	81(3)
C32	-841(4)	7375(7)	3234.5(19)	83(3)
C33	122(5)	6763(7)	3074.9(15)	78(2)
C34	983(4)	6640(7)	3462(2)	90(3)
C35	882(4)	7128(7)	4009.0(19)	81(3)
C36A	1902(9)	7000(20)	4381(6)	109(5)
C36B	-1998(17)	8660(40)	3840(14)	109(5)
C37	220(7)	6303(11)	2480(2)	88(3)
C38	-662(7)	6472(14)	2029(3)	100(3)
C39	-8(7)	6151(7)	1516(3)	104(4)
C40	-502(8)	4908(9)	1177(4)	132(5)
C41	137(5)	7465(6)	1147(2)	89(3)
C42	-717(3)	8403(8)	1031(3)	105(3)
C43	-566(5)	9622(7)	702(3)	101(3)
C44	437(6)	9902(7)	490(3)	108(4)
C45	1291(4)	8964(9)	605(3)	110(4)
C46	1141(4)	7745(7)	934(3)	102(4)

Table S4. Anisotropic Displacement Parameters (Å^2×10^3) for CMV280.

Atom	U_{11}	U_{22}	U_{33}	U_{23}	U_{13}	U_{12}
Cl1	502(15)	162(5)	256(8)	-92(6)	-25(9)	82(7)
Cl2	224(5)	289(8)	159(4)	-82(5)	-72(4)	50(5)
S1	92.2(16)	93.0(16)	62.0(12)	1.7(12)	-16.6(11)	4.2(14)
F1	263(12)	279(15)	115(7)	17(8)	2(7)	-126(12)
F2	240(11)	268(14)	101(6)	19(8)	46(7)	-6(11)
F3	309(13)	149(8)	101(6)	51(6)	-8(7)	-18(8)
O1	93(5)	135(6)	81(4)	20(4)	-2(4)	25(5)
O2	147(7)	114(6)	86(4)	-6(4)	-37(4)	-19(5)
O3	144(6)	62(4)	73(4)	-4(3)	-22(4)	13(4)
O4	178(8)	146(8)	69(4)	-8(5)	13(5)	42(7)
N1	118(6)	67(4)	51(4)	1(3)	-12(4)	20(4)
N2	148(9)	136(9)	75(5)	6(6)	-2(6)	27(8)
C1	130(9)	117(9)	69(6)	-4(6)	-10(6)	-12(8)
C2	95(7)	84(6)	63(5)	-6(4)	-12(5)	4(5)
C3	107(7)	60(5)	65(5)	7(4)	-10(5)	-2(5)
C4	116(8)	76(6)	54(4)	-2(4)	-10(5)	7(5)
C5	124(8)	79(6)	56(5)	-1(4)	-12(5)	-2(6)
C6	94(6)	60(5)	60(4)	5(4)	-20(4)	-1(4)
C7	104(7)	63(5)	54(4)	9(4)	-2(4)	-8(5)
C8	98(7)	65(5)	63(5)	7(4)	-10(4)	-7(4)
C9	95(6)	80(6)	61(5)	8(5)	-17(4)	10(5)
C10	111(8)	82(6)	61(5)	12(5)	-6(5)	-22(6)
C11	103(7)	76(6)	63(5)	-6(4)	4(5)	2(5)
C12	106(7)	69(6)	62(5)	7(4)	-12(5)	-1(5)
C13A	91(8)	97(9)	80(7)	21(6)	2(6)	-1(7)

Table S4. Anisotropic Displacement Parameters (Å^2×10^3) for CMV280.

Atom	U_{11}	U_{22}	U_{33}	U_{23}	U_{13}	U_{12}
C13B	91(8)	97(9)	80(7)	21(6)	2(6)	-1(7)
C14	131(9)	87(7)	63(5)	13(5)	5(5)	3(6)
C15	135(9)	88(7)	61(5)	6(5)	-7(5)	-5(6)
C16	146(11)	150(12)	60(6)	18(7)	11(6)	28(9)
C17	230(20)	177(17)	77(8)	5(10)	13(11)	-65(16)
C18	166(12)	126(10)	60(5)	-10(6)	3(7)	21(9)
C19	137(11)	130(11)	99(8)	-27(8)	-20(8)	23(9)
C20	189(16)	169(16)	109(10)	-47(11)	-6(10)	1(13)
C21	250(20)	167(18)	165(17)	-107(15)	12(15)	1(16)
C22	290(30)	118(13)	141(15)	-41(12)	13(16)	35(16)
C23	300(20)	138(14)	89(9)	-11(9)	8(12)	59(15)
Cl3	123(2)	201(4)	95(2)	33(2)	-4.8(17)	7(3)
Cl4	161(3)	155(3)	109(2)	23(2)	-14(2)	42(3)
S2	103.9(19)	134(2)	60.3(12)	3.6(14)	-18.2(13)	4.4(18)
F4	244(9)	131(6)	73(4)	-28(4)	5(5)	0(6)
F5	201(9)	173(8)	88(5)	-23(5)	-22(5)	-53(7)
F6	343(14)	128(7)	94(5)	-15(5)	23(7)	-41(8)
O5	164(8)	171(9)	79(5)	-1(6)	-44(5)	-55(7)
O6	130(7)	231(12)	74(4)	6(6)	3(4)	67(8)
O7	168(7)	56(4)	62(3)	-3(3)	-23(4)	7(4)
O8	179(8)	124(6)	54(3)	4(4)	-3(4)	26(6)
N3	164(8)	60(5)	50(4)	0(3)	-26(4)	1(5)
N4	142(8)	98(6)	61(4)	4(4)	-19(5)	16(6)
C24	146(10)	121(10)	74(6)	31(7)	-18(6)	18(8)
C25	111(7)	73(6)	56(5)	3(4)	-16(5)	-6(5)

Table S4. Anisotropic Displacement Parameters (Å^2×10^3) for CMV280.

Atom	U_{11}	U_{22}	U_{33}	U_{23}	U_{13}	U_{12}
C26	131(9)	80(6)	51(4)	7(4)	-13(5)	-21(6)
C27	155(10)	61(5)	52(4)	-3(4)	-17(5)	6(5)
C28	156(10)	82(7)	66(5)	-4(5)	-15(6)	-32(7)
C29	117(7)	69(6)	54(4)	-4(4)	-17(5)	3(5)
C30	119(8)	52(5)	54(4)	1(3)	-8(5)	-3(5)
C31	117(8)	67(5)	58(5)	5(4)	-14(5)	11(5)
C32	116(8)	75(6)	54(4)	6(4)	-22(5)	-13(6)
C33	108(7)	69(5)	55(4)	5(4)	-11(5)	-2(5)
C34	128(9)	84(7)	58(5)	17(5)	-7(5)	3(6)
C35	100(7)	82(6)	60(5)	6(5)	-17(5)	-15(5)
C36A	124(12)	121(13)	79(8)	-18(9)	-28(8)	5(10)
C36B	124(12)	121(13)	79(8)	-18(9)	-28(8)	5(10)
C37	120(9)	82(6)	60(5)	10(4)	-7(5)	7(6)
C38	139(9)	113(9)	48(4)	-7(5)	-6(5)	18(7)
C39	136(10)	123(9)	51(5)	-2(5)	-12(5)	22(8)
C40	199(16)	135(13)	62(7)	-12(7)	-1(8)	-30(11)
C41	87(6)	126(9)	52(4)	-1(5)	-15(4)	12(6)
C42	124(9)	126(9)	62(5)	5(6)	-14(5)	-1(8)
C43	93(8)	135(10)	74(6)	5(6)	-8(5)	7(7)
C44	138(10)	119(9)	66(6)	10(6)	-19(6)	17(8)
C45	107(8)	161(12)	62(5)	2(7)	-11(5)	23(8)
C46	103(8)	140(10)	62(5)	-14(6)	-18(5)	33(7)

Table S5. Bond Lengths for CMV280.

Atom	Atom	Length/Å	Atom	Atom	Length/Å
Cl1	C22	1.731(4)	Cl3	C45	1.725(4)
Cl2	C20	1.726(4)	Cl4	C43	1.730(3)
S1	O1	1.381(8)	S2	O5	1.431(10)
S1	O2	1.442(8)	S2	O6	1.407(9)
S1	C1	1.755(5)	S2	C24	1.760(5)
S1	C2	1.759(5)	S2	C25	1.759(5)
F1	C17	1.332(5)	F4	C40	1.326(5)
F2	C17	1.333(5)	F5	C40	1.326(5)
F3	C17	1.332(5)	F6	C40	1.326(5)
O3	C6	1.220(5)	O7	C29	1.215(5)
O4	N2	1.404(11)	O8	N4	1.441(10)
O4	C16	1.434(5)	O8	C39	1.428(5)
N1	C4	1.469(5)	N3	C27	1.462(5)
N1	C6	1.351(5)	N3	C29	1.346(5)
N2	C14	1.277(5)	N4	C37	1.276(5)
C2	C3	1.504(5)	C25	C26	1.522(5)
C2	C5	1.509(5)	C25	C28	1.525(5)
C3	C4	1.521(5)	C26	C27	1.505(5)
C4	C5	1.527(5)	C27	C28	1.518(5)
C6	C7	1.487(8)	C29	C30	1.502(5)
C7	C8	1.3900	C30	C31	1.3900
C7	C12	1.3900	C30	C35	1.3900
C8	C9	1.3900	C31	C32	1.3900
C8	C13B	1.518(6)	C31	C36B	1.519(6)
C9	C10	1.3900	C32	C33	1.3900

Table S5. Bond Lengths for CMV280.

Atom	Atom	Length/Å	Atom	Atom	Length/Å
C10	C11	1.3900	C33	C34	1.3900
C10	C14	1.491(5)	C33	C37	1.491(5)
C11	C12	1.3900	C34	C35	1.3900
C12	C13A	1.520(5)	C35	C36A	1.523(5)
C14	C15	1.495(5)	C37	C38	1.512(5)
C15	C16	1.510(5)	C38	C39	1.525(5)
C16	C17	1.499(5)	C39	C40	1.521(5)
C16	C18	1.502(5)	C39	C41	1.516(5)
C18	C19	1.3900	C41	C42	1.3900
C18	C23	1.3900	C41	C46	1.3900
C19	C20	1.3900	C42	C43	1.3900
C20	C21	1.3900	C43	C44	1.3900
C21	C22	1.3900	C44	C45	1.3900
C22	C23	1.3900	C45	C46	1.3900

Table S6. Bond Angles for CMV280.

Atom	Atom	Atom	Angle/°	Atom	Atom	Atom	Angle/°
O1	S1	O2	116.9(5)	O5	S2	C24	108.8(6)
O1	S1	C1	108.9(6)	O5	S2	C25	106.7(5)
O1	S1	C2	108.2(4)	O6	S2	O5	117.6(7)
O2	S1	C1	108.8(5)	O6	S2	C24	111.0(7)
O2	S1	C2	108.8(4)	O6	S2	C25	109.0(4)
C1	S1	C2	104.6(5)	C25	S2	C24	102.6(5)
N2	O4	C16	108.6(7)	C39	O8	N4	109.3(7)
C6	N1	C4	120.8(7)	C29	N3	C27	120.1(6)
C14	N2	O4	109.1(7)	C37	N4	O8	107.7(7)
C3	C2	S1	117.5(5)	C26	C25	S2	119.6(6)
C3	C2	C5	89.7(6)	C26	C25	C28	87.6(6)
C5	C2	S1	120.4(6)	C28	C25	S2	115.1(6)
C2	C3	C4	87.1(5)	C27	C26	C25	88.0(5)
N1	C4	C3	121.5(7)	N3	C27	C26	117.6(6)
N1	C4	C5	117.5(6)	N3	C27	C28	119.7(8)
C3	C4	C5	88.4(5)	C26	C27	C28	88.5(6)
C2	C5	C4	86.7(5)	C27	C28	C25	87.5(5)
O3	C6	N1	121.4(7)	O7	C29	N3	123.4(6)
O3	C6	C7	122.1(6)	O7	C29	C30	120.6(6)
N1	C6	C7	116.5(6)	N3	C29	C30	116.1(5)
C8	C7	C6	116.3(5)	C31	C30	C29	117.1(5)
C8	C7	C12	120.0	C31	C30	C35	120.0
C12	C7	C6	123.7(5)	C35	C30	C29	122.9(5)
C7	C8	C13B	126.6(19)	C30	C31	C32	120.0
C9	C8	C7	120.0	C30	C31	C36B	128.6(12)

Table S6. Bond Angles for CMV280.

Atom	Atom	Atom	Angle/°	Atom	Atom	Atom	Angle/°
C9	C8	C13B	113.1(19)	C32	C31	C36B	110.8(12)
C8	C9	C10	120.0	C33	C32	C31	120.0
C9	C10	C11	120.0	C32	C33	C34	120.0
C9	C10	C14	121.1(5)	C32	C33	C37	118.7(5)
C11	C10	C14	118.9(5)	C34	C33	C37	121.2(5)
C12	C11	C10	120.0	C35	C34	C33	120.0
C7	C12	C13A	123.4(7)	C30	C35	C36A	125.3(7)
C11	C12	C7	120.0	C34	C35	C30	120.0
C11	C12	C13A	116.5(7)	C34	C35	C36A	114.6(7)
N2	C14	C10	120.0(7)	N4	C37	C33	119.9(7)
N2	C14	C15	113.7(7)	N4	C37	C38	115.8(6)
C10	C14	C15	126.0(7)	C33	C37	C38	124.2(6)
C14	C15	C16	99.2(6)	C37	C38	C39	98.9(5)
O4	C16	C15	105.5(7)	O8	C39	C38	106.9(6)
O4	C16	C17	105.8(8)	O8	C39	C40	103.6(7)
O4	C16	C18	107.9(7)	O8	C39	C41	111.5(6)
C17	C16	C15	114.5(8)	C40	C39	C38	110.9(7)
C17	C16	C18	107.4(7)	C41	C39	C38	112.9(7)
C18	C16	C15	115.2(7)	C41	C39	C40	110.6(6)
F1	C17	F2	105.7(11)	F4	C40	C39	111.5(7)
F1	C17	F3	107.4(11)	F5	C40	F4	106.5(9)
F1	C17	C16	110.2(9)	F5	C40	F6	106.6(10)
F2	C17	C16	112.6(9)	F5	C40	C39	111.5(7)
F3	C17	F2	109.0(11)	F6	C40	F4	107.6(9)
F3	C17	C16	111.6(9)	F6	C40	C39	112.7(7)

Table S6. Bond Angles for CMV280.

Atom	Atom	Atom	Angle/°	Atom	Atom	Atom	Angle/°
C19	C18	C16	122.1(7)	C42	C41	C39	120.6(6)
C19	C18	C23	120.0	C42	C41	C46	120.0
C23	C18	C16	117.9(7)	C46	C41	C39	119.4(6)
C20	C19	C18	120.0	C41	C42	C43	120.0
C19	C20	Cl2	120.9(7)	C42	C43	Cl4	121.8(5)
C19	C20	C21	120.0	C44	C43	Cl4	118.2(5)
C21	C20	Cl2	119.0(7)	C44	C43	C42	120.0
C22	C21	C20	120.0	C45	C44	C43	120.0
C21	C22	Cl1	114.3(8)	C44	C45	Cl3	118.2(5)
C21	C22	C23	120.0	C44	C45	C46	120.0
C23	C22	Cl1	125.4(8)	C46	C45	Cl3	121.8(5)
C22	C23	C18	120.0	C45	C46	C41	120.0

Table S7. Hydrogen Bonds for CMV280.

D H A	d(D-H)/Å	d(H-A)/Å	d(D-A)/Å	D-H-A/°
N1 H1 O3[1]	0.88	1.99	2.813(9)	156.1
N3 H3 O7[2]	0.88	2.00	2.828(9)	157.1

[1]1-*x*, 1/2+*y*, -*z*; [2]-*x*, -1/2+*y*, 1-*z*

Table S8. Torsion Angles for mCMV280.

A	B	C	D	Angle/°	A	B	C	D	Angle/°
Cl1	C22	C23	C18	174.3(12)	Cl3	C45	C46	C41	179.7(6)
Cl2	C20	C21	C22	-178.4(9)	Cl4	C43	C44	C45	178.5(5)
S1	C2	C3	C4	-146.2(6)	S2	C25	C26	C27	-139.4(7)
S1	C2	C5	C4	143.7(7)	S2	C25	C28	C27	143.3(6)
O1	S1	C2	C3	50.8(8)	O5	S2	C25	C26	177.6(7)
O1	S1	C2	C5	-56.6(9)	O5	S2	C25	C28	75.4(8)
O2	S1	C2	C3	-77.1(8)	O6	S2	C25	C26	49.7(9)
O2	S1	C2	C5	175.5(8)	O6	S2	C25	C28	-52.6(9)
O3	C6	C7	C8	44.6(11)	O7	C29	C30	C31	-45.1(12)
O3	C6	C7	C12	-133.6(8)	O7	C29	C30	C35	132.3(8)
O4	N2	C14	C10	179.4(9)	O8	N4	C37	C33	-174.4(8)
O4	N2	C14	C15	-6.4(15)	O8	N4	C37	C38	1.9(14)
O4	C16	C17	F1	58.4(12)	O8	C39	C40	F4	-60.8(9)
O4	C16	C17	F2	-59.3(11)	O8	C39	C40	F5	-179.7(7)
O4	C16	C17	F3	177.6(9)	O8	C39	C40	F6	60.4(10)
O4	C16	C18	C19	-177.3(7)	O8	C39	C41	C42	-162.5(5)
O4	C16	C18	C23	1.2(10)	O8	C39	C41	C46	16.0(8)
N1	C4	C5	C2	-146.3(8)	N3	C27	C28	C25	-143.0(7)
N1	C6	C7	C8	-134.1(7)	N3	C29	C30	C31	133.9(8)
N1	C6	C7	C12	47.7(10)	N3	C29	C30	C35	-48.7(11)
N2	O4	C16	C15	17.1(12)	N4	O8	C39	C38	-11.5(11)
N2	O4	C16	C17	-104.6(10)	N4	O8	C39	C40	-128.6(8)
N2	O4	C16	C18	140.7(9)	N4	O8	C39	C41	112.3(8)
N2	C14	C15	C16	16.1(13)	N4	C37	C38	C39	-8.4(13)
C1	S1	C2	C3	166.8(7)	C24	S2	C25	C26	-68.1(9)

Table S8. Torsion Angles for mCMV280.

A	B	C	D	Angle/˚	A	B	C	D	Angle/˚
C1	S1	C2	C5	59.4(9)	C24	S2	C25	C28	-170.3(8)
C2	C3	C4	N1	142.9(7)	C25	C26	C27	N3	144.9(9)
C2	C3	C4	C5	21.3(7)	C25	C26	C27	C28	21.9(8)
C3	C2	C5	C4	21.5(7)	C26	C25	C28	C27	21.6(8)
C3	C4	C5	C2	-21.3(7)	C26	C27	C28	C25	-21.8(8)
C4	N1	C6	O3	-0.1(14)	C27	N3	C29	O7	0.7(16)
C4	N1	C6	C7	178.6(7)	C27	N3	C29	C30	-178.3(8)
C5	C2	C3	C4	-21.6(7)	C28	C25	C26	C27	-21.8(8)
C6	N1	C4	C3	95.3(10)	C29	N3	C27	C26	158.9(9)
C6	N1	C4	C5	-158.4(9)	C29	N3	C27	C28	-95.9(11)
C6	C7	C8	C9	-178.3(6)	C29	C30	C31	C32	177.6(6)
C6	C7	C8	C13B	7(3)	C29	C30	C31	C36B	7(2)
C6	C7	C12	C11	178.2(6)	C29	C30	C35	C34	-177.4(7)
C6	C7	C12	C13A	3.3(9)	C29	C30	C35	C36A	-0.8(12)
C7	C8	C9	C10	0.0	C30	C31	C32	C33	0.0
C8	C7	C12	C11	0.0	C31	C30	C35	C34	0.0
C8	C7	C12	C13A	-174.9(8)	C31	C30	C35	C36A	176.6(11)
C8	C9	C10	C11	0.0	C31	C32	C33	C34	0.0
C8	C9	C10	C14	-179.3(7)	C31	C32	C33	C37	-177.6(7)
C9	C10	C11	C12	0.0	C32	C33	C34	C35	0.0
C9	C10	C14	N2	160.1(9)	C32	C33	C37	N4	176.6(8)
C9	C10	C14	C15	-13.2(13)	C32	C33	C37	C38	0.7(13)
C10	C11	C12	C7	0.0	C33	C34	C35	C30	0.0
C10	C11	C12	C13A	175.3(8)	C33	C34	C35	C36A	-177.0(10)
C10	C14	C15	C16	-170.1(9)	C33	C37	C38	C39	167.7(9)

Table S8. Torsion Angles for mCMV280.

A	B	C	D	Angle/°	A	B	C	D	Angle/°
C11	C10	C14	N2	-19.2(13)	C34	C33	C37	N4	-0.9(13)
C11	C10	C14	C15	167.4(9)	C34	C33	C37	C38	-176.9(9)
C12	C7	C8	C9	0.0	C35	C30	C31	C32	0.0
C12	C7	C8	C13B	-174(3)	C35	C30	C31	C36B	-171(2)
C13B	C8	C9	C10	175(2)	C36B	C31	C32	C33	172.4(19)
C14	C10	C11	C12	179.4(7)	C37	C33	C34	C35	177.6(7)
C14	C15	C16	O4	-18.8(10)	C37	C38	C39	O8	11.3(10)
C14	C15	C16	C17	97.1(9)	C37	C38	C39	C40	123.6(9)
C14	C15	C16	C18	-137.6(8)	C37	C38	C39	C41	-111.7(8)
C15	C16	C17	F1	-57.3(13)	C38	C39	C40	F4	-175.1(8)
C15	C16	C17	F2	-175.0(8)	C38	C39	C40	F5	66.0(9)
C15	C16	C17	F3	62.0(11)	C38	C39	C40	F6	-53.9(11)
C15	C16	C18	C19	-59.9(11)	C38	C39	C41	C42	-42.2(8)
C15	C16	C18	C23	118.7(8)	C38	C39	C41	C46	136.4(6)
C16	O4	N2	C14	-7.1(14)	C39	O8	N4	C37	6.3(12)
C16	C18	C19	C20	178.5(9)	C39	C41	C42	C43	178.5(6)
C16	C18	C23	C22	-178.6(9)	C39	C41	C46	C45	-178.5(6)
C17	C16	C18	C19	69.0(10)	C40	C39	C41	C42	82.8(7)
C17	C16	C18	C23	-112.5(8)	C40	C39	C41	C46	-98.7(7)
C18	C16	C17	F1	173.4(11)	C41	C39	C40	F4	58.8(10)
C18	C16	C17	F2	55.7(11)	C41	C39	C40	F5	-60.1(9)
C18	C16	C17	F3	-67.3(11)	C41	C39	C40	F6	-180.0(9)
C18	C19	C20	Cl2	178.3(9)	C41	C42	C43	Cl4	-178.4(6)
C18	C19	C20	C21	0.0	C41	C42	C43	C44	0.0
C19	C18	C23	C22	0.0	C42	C41	C46	C45	0.0

Table S8. Torsion Angles for mCMV280.

A	B	C	D	Angle/°	A	B	C	D	Angle/°
C19	C20	C21	C22	0.0	C42	C43	C44	C45	0.0
C20	C21	C22	Cl1	-174.9(11)	C43	C44	C45	Cl3	-179.7(6)
C20	C21	C22	C23	0.0	C43	C44	C45	C46	0.0
C21	C22	C23	C18	0.0	C44	C45	C46	C41	0.0
C23	C18	C19	C20	0.0	C46	C41	C42	C43	0.0

Table S9. Hydrogen Atom Coordinates (Å×10^4) and Isotropic Displacement Parameters (Å^2×10^3) for mCMV280.

Atom	*x*	*y*	*z*	U(eq)
H1	4614	4257	-110	95
H1A	5031	4305	-2591	159
H1B	4378	5632	-2342	159
H1C	3919	4811	-2892	159
H2	5123	2335	-1761	97
H3A	4215	1097	-1103	93
H3B	3418	2459	-981	93
H4	5714	2280	-745	99
H5A	4663	4909	-1134	104
H5B	5842	4299	-1303	104
H8	6515	2592	1137	91
H9	6346	3472	2050	95
H11	3318	4837	1637	97
H12	3487	3957	725	95
H13A	3160	4736	333	134
H13B	2439	4149	826	134
H13C	2970	3036	404	134
H13D	6989	2043	758	134
H13E	7177	1732	1415	134
H13F	7506	3264	1161	134
H15A	6123	5006	2982	114
H15B	5530	3469	3066	114
H19	6362	4455	4198	147
H21	6892	8353	4934	232
H23	4557	7914	3710	210

Table S9. Hydrogen Atom Coordinates (Å×10^4) and Isotropic Displacement Parameters (Å^2×10^3) for mCMV280.

Atom	*x*	*y*	*z*	U(eq)
H3	385	6649	5114	111
H24A	1380	5854	7824	171
H24B	539	5399	7329	171
H24C	306	6781	7709	171
H25	-29	8501	6801	97
H26A	381	5970	6123	105
H26B	-787	6553	6318	105
H27	-716	8580	5772	108
H28A	809	9766	6100	122
H28B	1594	8408	5958	122
H31	-1600	8281	3890	97
H32	-1429	7459	2970	99
H34	1641	6222	3353	108
H35	1471	7044	4274	97
H36A	1949	7824	4640	163
H36B	2532	6997	4147	163
H36C	1883	6100	4597	163
H36D	-2397	8232	4143	163
H36E	-2427	8597	3487	163
H36F	-1850	9683	3926	163
H38A	-963	7463	2019	120
H38B	-1250	5763	2073	120
H42	-1403	8212	1176	126
H44	540	10735	265	130
H46	1724	7103	1013	123

Table S10. Atomic Occupancy for CMV280.

Atom	*Occupancy*	Atom	*Occupancy*	Atom	*Occupancy*
H8	0.791(14)	H12	0.209(14)	C13A	0.791(14)
H13A	0.791(14)	H13B	0.791(14)	H13C	0.791(14)
C13B	0.209(14)	H13D	0.209(14)	H13E	0.209(14)
H13F	0.209(14)	H31	0.685(14)	H35	0.315(14)
C36A	0.685(14)	H36A	0.685(14)	H36B	0.685(14)
H36C	0.685(14)	C36B	0.315(14)	H36D	0.315(14)
H36E	0.315(14)	H36F	0.315(14)		

Experimental

Single crystal X-ray diffraction data of **mCMV280** was collected at 180 K on a Rigaku XtaLAB Synergy-DW diffractometer, with Cu Kα radiation (λ = 1.54184 Å). Data reduction and empirical absorption correction were performed using the CrysAlisPro program. The structure was solved by a dual-space algorithm using SHELXT program. All non-hydrogen atoms could be located directly from the difference Fourier maps. Framework hydrogen atoms were placed geometrically and constrained using the riding model to the parent atoms. Final structure refinement was done using the SHELXL program by minimizing the sum of squared deviations of F^2 using a full-matrix technique.

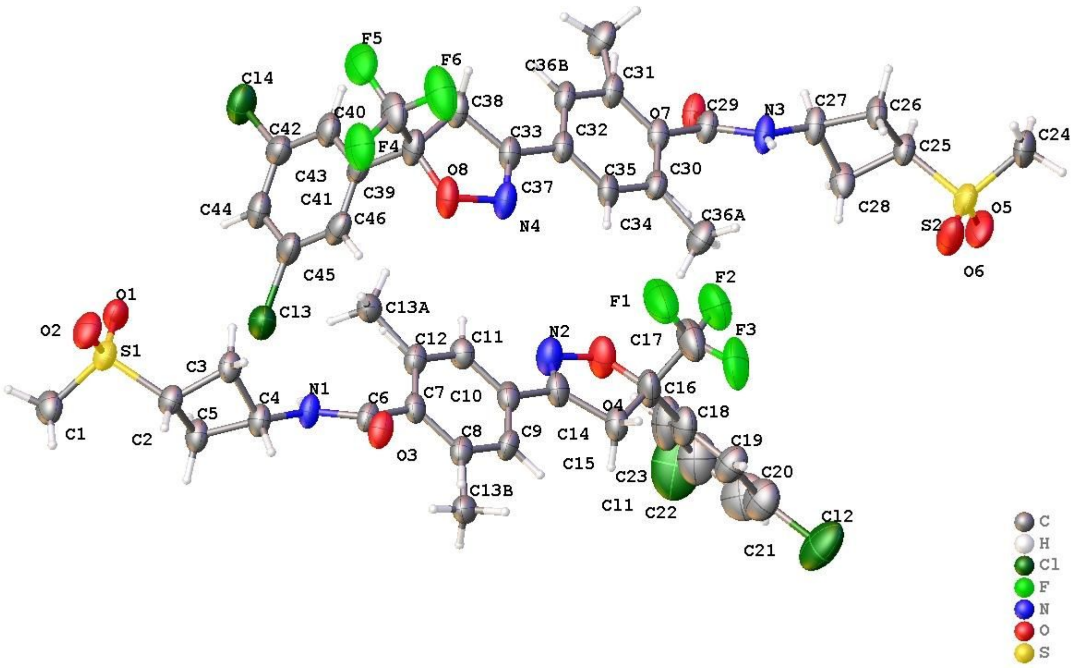

Figure S25. Single crystal X-ray diffraction data of mCMV280

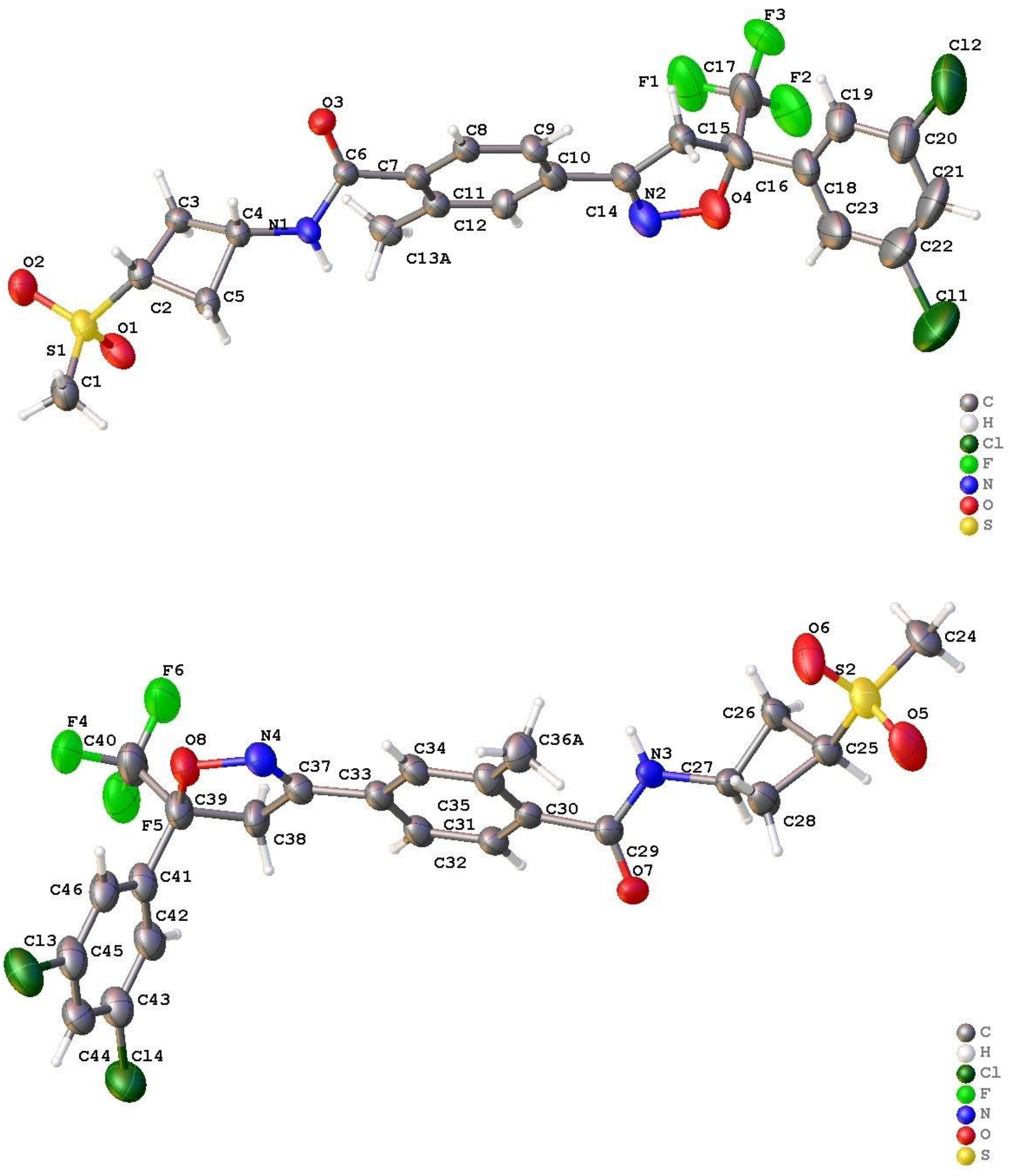

Figure S26. Single crystal X-ray diffraction data of mCMV280
